# Transcriptomic responses of *Saccharum spontaneum* roots in response to polyethylene glycol – 6000 stimulated drought stress

**DOI:** 10.3389/fpls.2022.992755

**Published:** 2022-10-24

**Authors:** Kai-Chao Wu, Cheng-Mei Huang, Krishan K. Verma, Zhi-Nian Deng, Hai-Rong Huang, Tian Pang, Hui-Qing Cao, Hai-Bin Luo, Sheng-Li Jiang, Lin Xu

**Affiliations:** ^1^ Sugarcane Research Institute, Guangxi Academy of Agricultural Sciences, Nanning, China; ^2^ Key Laboratory of Sugarcane Biotechnology and Genetic Improvement (Guangxi), Ministry of Agriculture and Rural Area, Nanning, China; ^3^ Guangxi Key Laboratory of Sugarcane Genetic Improvement, Nanning, China; ^4^ Guangxi Crop Genetic Improvement and Biotechnology Laboratory, Nanning, China

**Keywords:** gene, transcriptome, signal transduction, drought stress, *Saccharum spontaneum*

## Abstract

Drought is the abiotic factor that adversely affects plant growth, development survival, and crop productivity, posing a substantial threat to sustainable agriculture worldwide, especially in warm and dry areas. However, the extent of damage depends upon the crop growth stage, severity and frequency of the stress. In general, the reproductive growth phase is more sensitive to stresses causing a substantial loss in crop productivity. *Saccharum spontaneum* (L.) is the most variable wild relative of sugarcane with potential for use in sugarcane crop improvement programs. In the present study addresses the transcriptomic analysis of drought stress imposed by polyethylene glycol-6000 (PED-6000; w/v- 25%) on the root tip tissues of *S. spontaneum* GX83-10. The analysis of microarrays of drought-stressed roots was performed at 0 (CK), 2 (T_2_), 4 (T_4_), 8 (T_8_) and 24 h (T_24_). The analyzed data were compared with the gene function annotations of four major databases, such as Nr, KOG/COG, Swiss-Prot, and KEGG, and a total of 62,988 single-gene information was obtained. The differently expressed genes of 56237 (T_4_), 59319 (T_8_), and 58583 (T_24_), among which CK obtained the most significant number of expressed genes (35920) as compared to T_24_, with a total of 53683 trend genes. Gene ontology (GO) and KEGG analysis were performed on the 6 important trends, and a total of 598 significant GO IDs and 42 significantly enriched metabolic pathways. Furthermore, these findings also aid in the selection of novel genes and promoters that can be used to potentially produce crop plants with enhanced stress resistance efficiency for sustainable agriculture.

## Introduction

Abiotic stress is a major problem to sustainable agriculture crop productivity around the globe, which can potentially affect the average crop production over 50% (Cui et al., 2017; Naeem et al., 2018; Waqas et al., 2019; Verma et al., 2021; Verma et al., 2022). Drought stress is one of the severe abiotic stress that inhibit plant growth, development and yield (Baiazidi-Aghdam et al., 2016). It is well-established that drought stress may impair various morpho-physiological, anatomical, cellular and metabolic processes associated with the regulation of plant development (Yoo et al., 2013; Liu et al., 2013; Cui et al., 2017). It also enhances leaf fall and chlorophyll damage, downregulate photosynthetic efficiency and loss in leaf area expansion/development, resulting in low plant productivity and sometimes total crop failure (Jiang et al., 2016; Kamran et al., 2018; Naeem et al., 2018; Verma et al., 2020; Verma et al., 2022). Polyethylene glycon (PEG) having molecular weight of 6000 is a natural polymer that is water soluble and nonionic. PEG 6000 is found to mimic drought stress and results in lowering of plant’s water potential due to osmotic stress (Ahmad et al., 2020).

During adverse environmental conditions, plants produce a large number of reactive oxygen species (ROS) associated with the regulation of various processes, i.e., pathogen defense, programmed cell death (PCD), and stomatal behavior (Liang et al., 2019). It causes oxidative damage to cell membranes, proteins, effective enzymatic activities [superoxide dismutase (SOD), catalase (CAT), peroxidase (POD), and ascorbate peroxidase (APX)], non-enzymatic (phenolic acids, carotenoids, flavonoids, ascorbic acid, proline, etc.), RNA and DNA (Dawood and El-Awadi, 2015; Baiazidi-Aghdam et al., 2016; Cui et al., 2017; Laxa et al., 2019; Verma et al., 2022). ROS plays important roles in maintaining normal plant growth, development and improving stress resistance capacity. The enhanced synthesis of osmolytes, i.e., soluble protein and proline, also plays stress tolerance functions (Sarropoulou et al., 2012; Turk et al., 2014). Cell death (also known as cell injury) is relative to the length or duration of exposure to stress and the severity of the damage caused (Turk et al., 2014; Verma et al., 2020).

*Saccharum spontaneum* L. is one of the wild species in the *Saccharum* complex and has a reservoir of genes for various economically important traits, such as tolerance to environmental stresses. Since the beginning of the new century, the world’s major sugarcane-producing countries have attached great importance to the investigation, collection, evaluation, and utilization of *S. spontaneum* germplasm resources (Zhang et al., 2010; Xu et al., 2017; Li et al., 2018; Xian et al., 2019; Xu et al., 2020; Xu et al., 2022). *S. spontaneum* has the most widely used wild germplasm for sugarcane breeding programs in China. In China, *S. spontaneum* grows in the wild and is widespread within the geographical range of northern latitudes (18-33°C) and eastern longitudes (97-122°C). More than 600 *S. spontaneum* clones were collected and conserved in China (Qi et al., 2022). Analysis of the genetic diversity of *S. spontaneum* using molecular markers showed significant geographical differences within the collection of *S. spontaneum* (Fan et al., 2013). It was obtained through interspecific hybridization combined with molecular markers to identify true hybrids and progeny and applied to sugarcane breeding (Yu et al., 2019; Zhang et al., 2019).

*Saccharum spontaneum* is most important for modern sugarcane breeding, commercial varieties contain about 10% of the chromosomes from *S. spontaneum*, and effectively used *S. spontaneum* stress-resistant parents and genes are minimal. At present, different drought stress resistance-related genes of *S. spontaneum* have been successfully cloned and preliminary functional analysis carried out, including SsPOD-1a, SsPOD-1b, SsSOD-1a, SsDREB2-a, SsDREB2-f, SsDREB2-1, SsDREB2-2, SsGST, SsCAT-1c, SsCAT-1d, Ssδ-OAT-2, SsREMO-1, ScPP2C, PIN gene family and NAC transcription factors (Xu et al., 2017; Liu et al., 2018; Yang et al., 2019; Huang et al., 2021; Qingqing et al., 2022). Assessing these efficient stress tolerance genes has provided new candidate gene resources for accelerating the genetic improvement of sugarcane. Still, limited reports were available on acquiring or utilizing significant stress tolerance genes in *S. spontaneum* for better crop improvement.

In the present study, based on the previous research (Wu et al., 2018a; Wu et al., 2018b), the stress-resistant genes were identified from the root system of *S. spontaneum* for further analysis of the regulation of molecular mechanisms in response to polyethylene glycol-6000 (PEG-6000; w/v 25%) stimulated drought stress and provide advanced approaches to improve the stress tolerance capacity by genetic engineering technologies.

## Materials and methods

### Plant material and experimental layout

The plant material of *S. spontaneum* GX83-10, the wild-type stress-resistant core germplasm, was collected in the Germplasm Resource Garden. The experiment was carried out in the greenhouse of Guangxi Sugarcane Genetic Improvement Key Laboratory, Sugarcane Research Institute, Guangxi Academy of Agricultural Sciences, Nanning, Guangxi, China. The pre-cultured seedlings with consistent rooting and growth were selected and transplanted (n = 50) in hydroponic culture with Hoagland nutrient solution (10 L). The nutrient solution was changed in a week at the seedling stage and every three days during elongation phase. The timing was set to oxygenate the nutrient solution once every hour during the experiment.

Each plant maintained 3 reproductive tiller stems during experimental duration, and juvenile tillers were removed regularly. The experiment was designed with five treatment levels, such as 0 h (CK), 2 h (T_2_), 4 h (T_4_), 8 h (T_8_), and 24 h (T_24_) under drought stress. Polyethylene glycol – 6000 (PEG-6000 w/v 25%) was used to stimulate drought stress treatment in *S. spontaneum* stems with five visible nodes. At the beginning of the experiment, root samples were collected at 0 h (T_0_, CK) treatment. PEG-6000 (25%) of the same volume was used to replace Hogland nutrient solution simultaneously, and root samples were collected 2, 4, 8 and 24 h after treatment. Three plants (*n* = 3) with consistent plant growth and proper root development were selected for transcriptomic analysis of each treatment. During sampling, ten white segments of root tips of each plant with a length of about 3-4 cm were cut and mixed. After naming, root samples were kept frozen under liquid N_2_ and stored at -80°C.

### Isolation, library construction and sequencing of RNA

Total RNA of CK, T_2_, T_4_, T_8_ and T_24_ was extracted with TRIzol reagent, respectively (Wu et al., 2018a). After total RNA extraction, eukaryotic mRNA was enriched by Oligo(dT) beads, while prokaryotic mRNA was enriched for removing rRNA by Ribo-Zero™ Magnetic Kit (Epicenter). The enriched mRNA was fragmented into short fragments using fragmentation buffer and reverse transcripted into cDNA with random primers. Second-strand of cDNA was synthesized by DNA polymerase-I, RNase H, dNTP and buffer. Then the cDNA fragments were purified with a QiaQuick PCR extraction kit, end-repaired, poly(A) added, and ligated to Illumina sequencing adapters. The ligation product size was selected by agarose gel electrophoresis, PCR amplified and sequenced using Illumina HiSeqTM 4000 by Gene Denovo Biotechnology Co. Guangzhou, China (**Supplementary Figure S1**). All sequencing reads were uploaded to the National Center for Biotechnology Information (NCBI) [SRA accession number – PRJNA835339].

### Transcriptome and reference transcriptome assembly

Filtering raw reads, *de novo* assembly was performed with short reads assembling program Trinity (Grabherr et al., 2011). The high-quality rRNA clean reads were removed and mapped to the reference transcriptome by default parameters of the short reads alignment tool Bowtie2 (Li et al., 2009) [**Figures S2** and **S3**].

### Analysis of unigene expression and basic annotation

The unigene expression was calculated and normalized to reads per kilobase per million (RPKM) (Mortazavi et al., 2008). The unigenes were annotated using the BLASTx program (http://www.ncbi.nlm.nih.gov/BLAST/) with an E-value threshold of 1e-5 to NCBI non-redundant protein sequence database (Nr) (http://www.ncbi.nlm.nih.gov), Swiss-Prot (http://www.expasy.ch/sprot), Kyoto Encyclopedia of Genes and Genomes (KEGG) (http://www.genome.jp/kegg), and COG/KOG (http://www.ncbi.nlm.nih.gov/COG). Protein functional annotations could be obtained according to the best alignment results.

### Identification and analysis of differentially expressed genes

The edgeR package (http://www.r-project.org/) was used to identify DEGs across samples or groups. Identified genes with fold change ≥2 and false discovery rate (FDR) ≤0.05 as significant DEGs. DEGs were subjected to enrichment analysis of GO functions and KEGG pathways (Xu et al., 2022). Gene expression pattern analysis was used to cluster genes of similar expression patterns for multiple samples (@ 3 times). To examine the expression pattern of DEGs, each sample was normalized to 0, log2 (v1/v0), log2 (v2/v0), and clustered by Short Time-series Expression Miner software (STEM) (Ernst and Bar-Joseph, 2006). The clustered profiles with p-value (≤ 0.05) were considered significant profiles. The DEGs in all or each profile were subjected to Gene Ontology (GO) and KEGG pathway enrichment analysis. The hypothesis of the p-value calculation and FDR correction showed that GO terms or pathways with Q value ≤ 0.05 were enriched GO terms or pathways (Benjamini and Hochberg, 1995).

### Quantitative real-time PCR (qRT-PCR) assay

Up-regulated genes were randomly selected for verification according to Wu et al. (2018a) and Xu et al. (2022). The validated samples, such as A_1_ (0 h), A_2_ (2 h), A_3_ (4 h), A_4_ (8 h) and A_5_ (24 h), treated with PEG-6000 (25%) for 0, 2, 4, 8 and 24 h, respectively. Unigene0049197 (alpha-tubulin) was used as a control gene. Special primers were designed for 13 different genes of expression validation of up-regulated genes using Premier 5.0 software (**Supplementary Table S1**).

## Results

**Figure 1:**
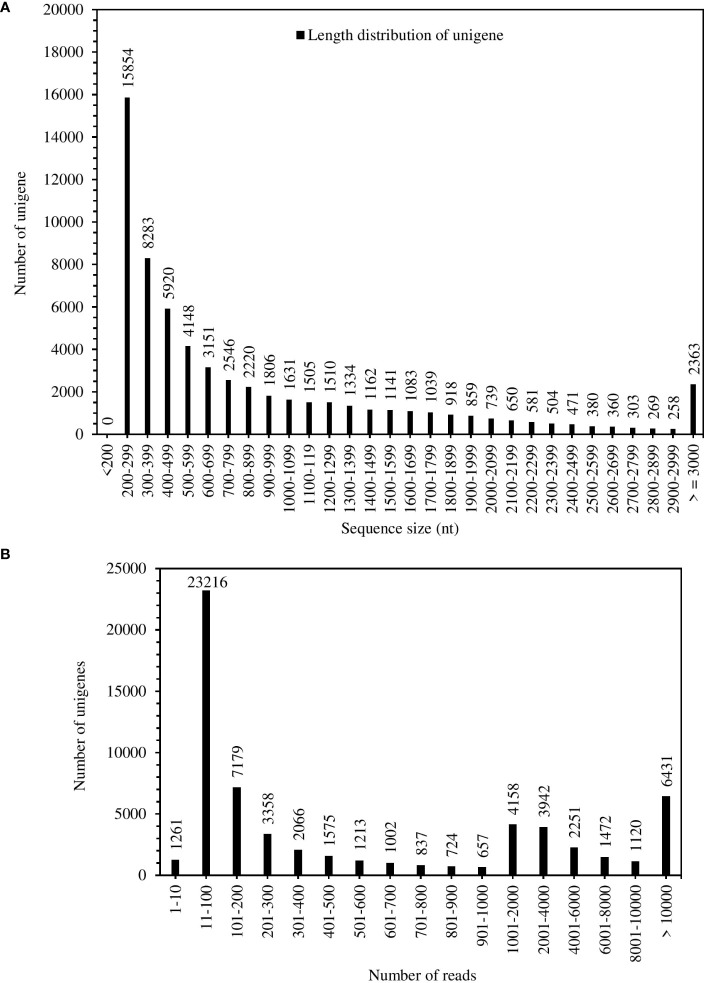
Unigenes length distribution **(A)** and coverage statistics **(B)** of *S. spontaneum*.

The total RNA quality was quantified by Agilent 2100 system (**Figure S4**). The results of total RNA extraction showed quality requirements for library construction (**Table S2**). A total of 57,036,239 bases were assembled in the experiment, and 62,988 genes were obtained (**Table S3**). The maximum length of the assembly (15429 nt), minimum (201 nt), average (905 nt) and N50 (1533 nt); the N50 length was larger than the average length (**Table S3**). **Figure 1A** showed the number of Unigenes of 1500 – 1599 nt was 1141, smaller than the number of genes of average length (1806). The unigenes have coverage distribution on reads of 1-1000 and above in *S. spontaneum*. Unigenes covering 11-100 reads were at 23216, followed by unigenes covering more than 1000 reads with 6431 (**Figure 1B**). Comprehensive analysis shows that the quality of the completed assembly was good.

### Analysis of gene annotation results

**Figure 2:**
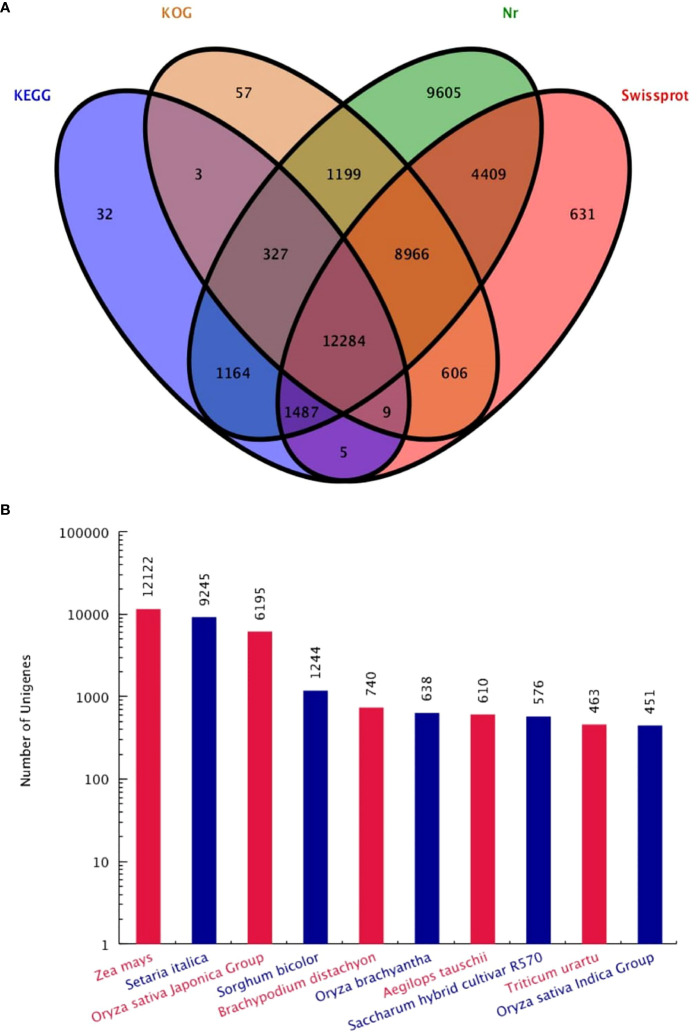
Venn diagram **(A)** of four major database annotations and statistical map of species distribution **(B)** of the first ten species are shown.

Functional annotation analysis of all 62,988 unigenes was found. The unigenes were matched as 39,411 (62.62%) in Nr database, 28,397 (45.08%) Swissprot database, 23,451 (36.65%) KOG database and 15,311 (24.31%) in the KEGG database. A total of 40,784 (64.75%) genes were successfully annotated, while 22,204 (35.25%) genes failed to be annotation (**Table 1**). The large number of unigenes were annotated with the Nr database, followed by Swissprot, KOG and KEGG (**Figure 2A**). Interestingly, KOG-Nr and Swissprot-Nr found the largest number of co-annotated unigenes. The Nr database revealed that 39,441 unigenes annotated sequences aligned with different plant species to known nucleotide sequences similar to other plants. Annotation results showed as 30.73, 23.44, 15.71, 3.15, 1.88, 1.62, 1.55, 1.46, 1.17 and 1.14%, matching with *Zea mays*, *Setaria italica*, *Oryza sativa* Japonica, *Sorghum bicolor*, *Brachypodium distachyon*, *Oryza brachyantha*, *Aegilops tauschii*, *Saccharum* hybrid cultivar R570, *Triticum urartu*, *Oryza sativa* Indica, respectively (**Figure 2B**). The results have high homology with *Zea mays, Setaria italica* and *Oryza sativa* Japonica genomes. These results support the genomic similarity of *S. spontaneum* tissues with the other plants, such as *Z. mays*, *O. sativa* and *S. bicolor*.

**Table 1:** Annotation statistics of four databases.

Total Unigenes	Nr	Swissprot	KOG	KEGG	annotation genes	without annotation genes
62988	39441	28397	23451	15311	40784	22204

### Gene ontology (GO) and KOG analysis

**Figure 3:**
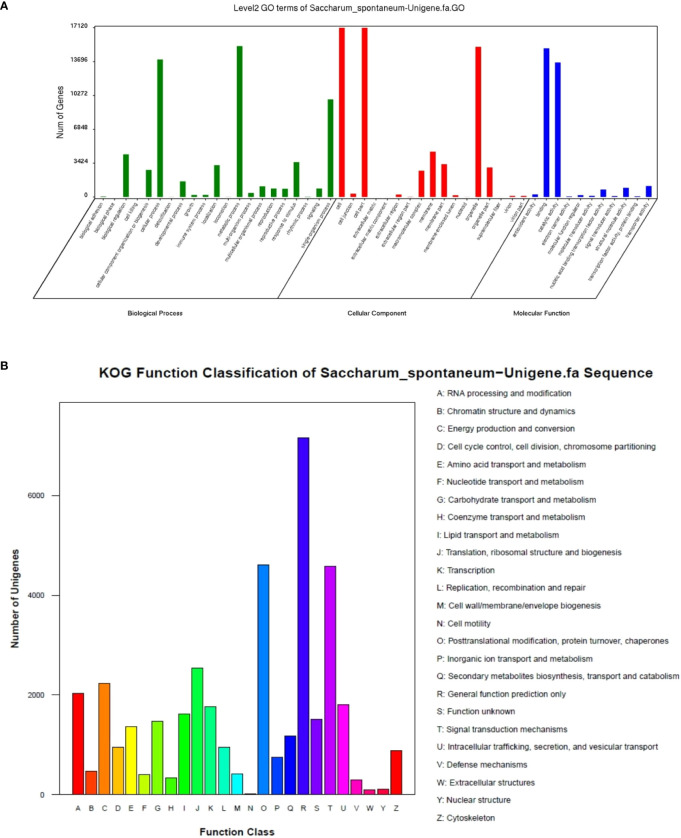
GO **(A)** and KOG **(B)** functions classification of expressed unigenes.

GO annotation found that all unigenes were enriched in 50 Ontology, 59093 unigenes (38.01%) in biological processes, 32282 (20.77%) in molecular functions, and 64072 (41.22%) in cellular components (**Figure 3A**). Most annotated classes in the biological process were included in “metabolic process” 15269 (9.82%), “cellular process” 13925 (8.96%), and “single-organism process” 9880 (6.36%). In the molecular function, most annotated unigenes were categorized as “binding” 15050 (9.68%), “catalytic activity” 13616 (8.76%), and “biological regulation” 4316 (2.78%). In the cellular component, most of the annotated unigenes were classified as “cell” 17120 (11.01%), “cell part” 17117 (11.01%), and “organelle” 15196 (9.78%). KOG classified all unigenes, and 39,559 unigenes were found to be aligned to the database and distributed into 25 categories (**Figure 3B**). KOG category as general function prediction was the largest group, i.e., 7,153 (18.08%), followed by post-translational modification, protein turnover, chaperones 4,602 (11.63%), and signal transduction mechanisms 4,576 (11.57%).

### KEGG analysis

Our data revealed that 9,056 unigenes were enriched in 130 pathways, and 4,818 (53.20%) unigenes annotated to ribosome of translation, carbon metabolism of global and overview, biosynthesis of amino acids of global and overview, plant-pathogen interaction of environmental adaptation, oxidative phosphorylation of energy metabolism, protein processing in the endoplasmic reticulum of folding, sorting and degradation, spliceosome of transcription, RNA transport of translation, purine metabolism of nucleotide metabolism and phenylpropanoid biosynthesis of other secondary metabolites. Ribosome 1,099 (12.14%) was the largest group, followed by carbon metabolism 652 (7.20%) and biosynthesis of amino acids 498 (5.50%), shown in **Table 2**.

**Table 2:** Pathway list of unigenes (only the first 10 rows).

Pathway ID	KEGG_B_class	Pathway	Unigene Count	Unigene Ratio (%)
ko03010	Translation	Ribosome	1099	12.14
ko01200	Global and Overview	Carbon metabolism	652	7.20
ko01230	Global and Overview	Biosynthesis of amino acids	498	5.50
ko04626	Environmental adaptation	Plant-pathogen interaction	482	5.32
ko00190	Energy metabolism	Oxidative phosphorylation	444	4.90
ko04141	Folding, sorting, and degradation	Protein processing in endoplasmic reticulum	402	4.44
ko03040	Transcription	Spliceosome	330	3.64
ko03013	Translation	RNA transport	314	3.47
ko00230	Nucleotide metabolism	Purine metabolism	300	3.31
ko00940	Biosynthesis of other secondary metabolites	Phenylpropanoid biosynthesis	297	3.28

### Changes in the expression and differentially expressed genes

The maximum number of genes was expressed in the T_8_ group, the minimum in T_0_ (CK), and the coverage of the total expressed genes of the samples were 99.16% (**Table 3**). **Figure S5** represents the log10FPKM value of each treatment is more significant than 2.5, and each sample’s corresponding gene expression levels were not much different. Each gene expression’s abundance value is higher than (T_1_) CK and T_8_ was highest.

**Table 3:** Genes expression statistics of each sample (total reference genes 62988).

Sample	genes Num	Ratio (%)	All samples genes	Ratio (%)
CK	55691	88.42	62461	99.16
T2	57455	91.22		
T4	56237	89.28		
T8	59319	94.18		
T24	58583	93.01		

**Figure 4:**
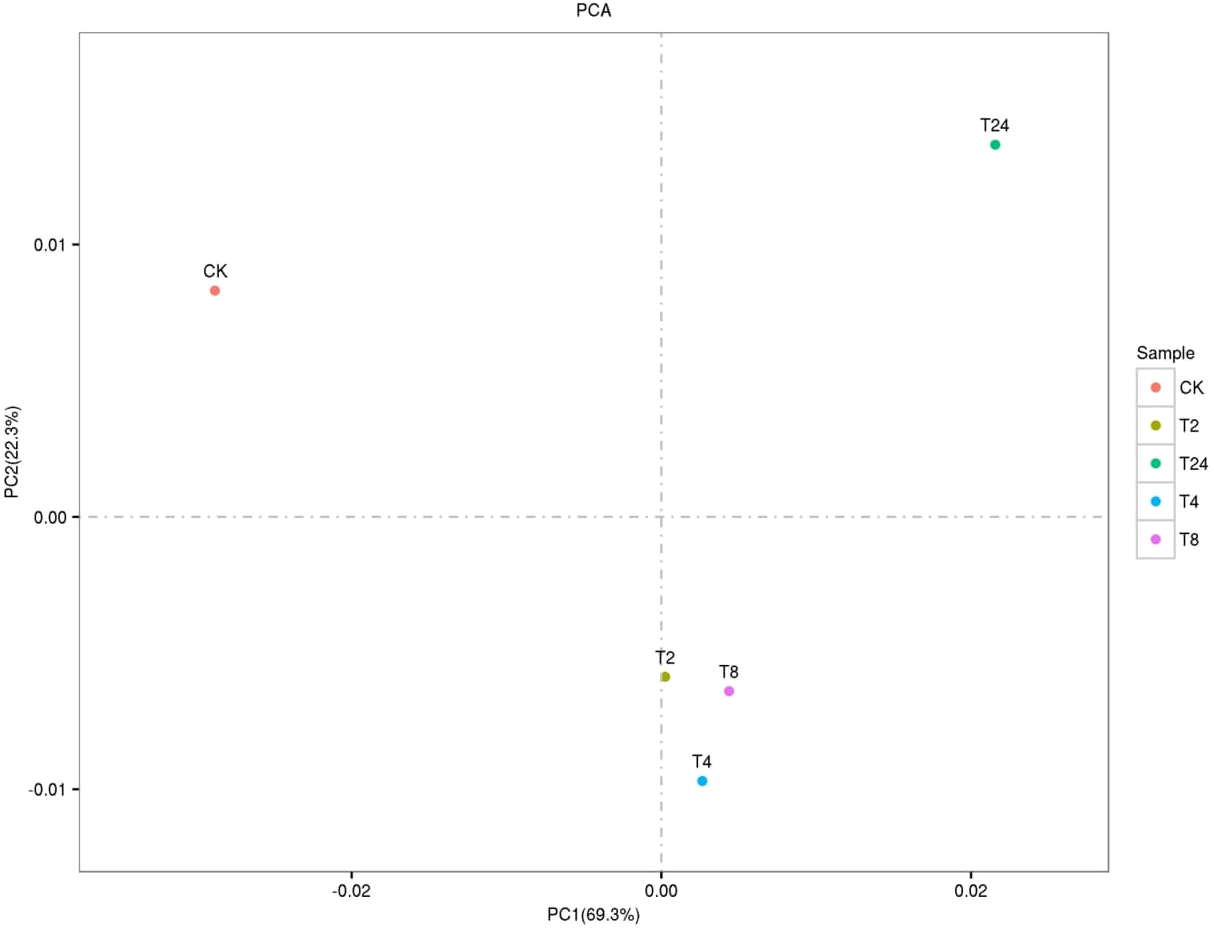
PCA two-dimensional coordinate diagram of six samples.

**Figure 5:**
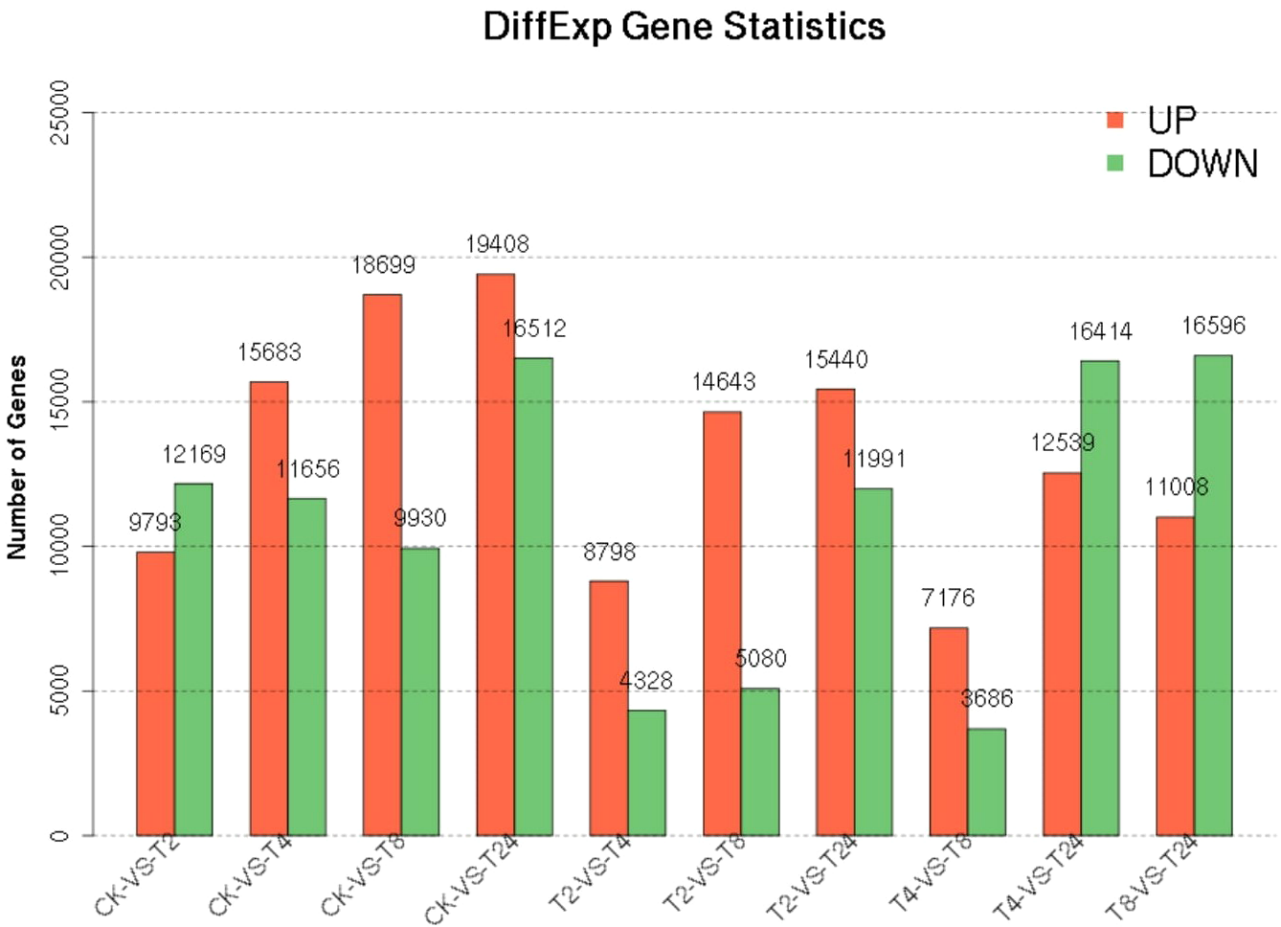
Statistics of differential genes between samples.

The PCA two-dimensional coordinate diagram shows that the PC1 explained 69.3% of the overall gene expression variance in all samples, and PC1 and PC2 explain 91.6% of the overall variance (**Figure 4**). As shown in **Figure S6**, the CK and treated plants are classified into two groups. The treated groups, such as T_2_, T_4,_ and T_8,_ belong to one subgroup, and T_24_ is a separate subgroup. The gene expression relationship between T_4_ and T_8_ treatments was more similar. The differentially expressed genes was compared among the samples FDR ≤ 0.05, and the difference fold was more than two times. () The number of upregulated (red dots), downregulated (green dots), and no difference (black dots) genes are shown in **Figure S7**. In terms of up-regulated genes, with the increase in processing time, the number of up-regulated genes among samples showed an increasing trend; group CK-vs-T_24_ showed the highest (19408) number of up-regulated genes (**Figure 5**). The number of down-regulated genes was comparatively low among T_2_, T_4_ and T_8_ as compared to T_24_. The number of down-regulated genes were high, especially T_4_-vs-T_24_ (16596) and T_8_-vs-T_24_ (16414) group, respectively. The down-regulated genes between CK and each treatment initially decreased and then increased, among which CK-vs-T_24_ showed that the number of down-regulated genes reached 16,512. The total number of DEGs, CK-vs-T_24,_ monitored the highest number of genes (35920) (**Figure S7**).

**Figure 6:**
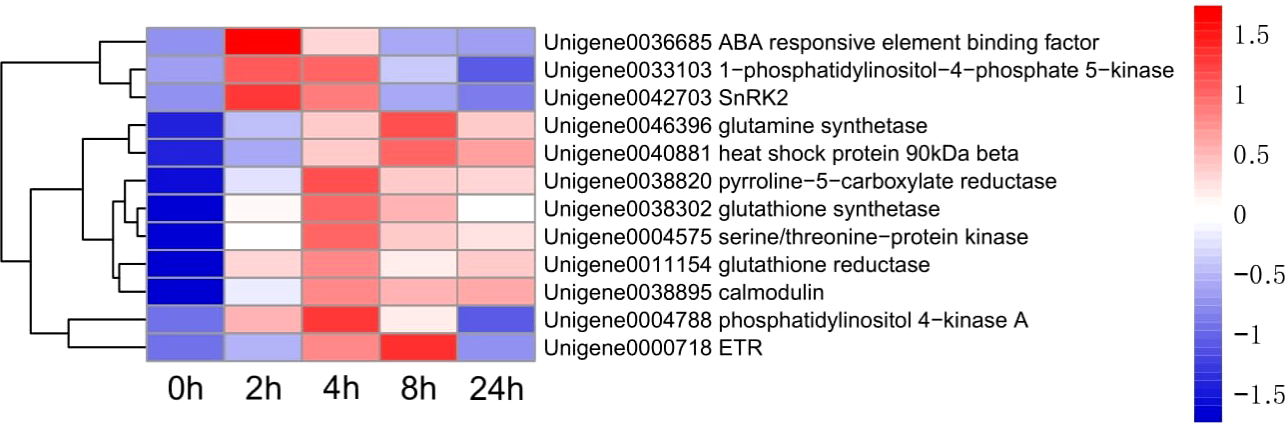
Heatmap of 12 genes expression.

### Gene ontology (GO) and differentially expressed genes (DEGs)

**Figure 7:**
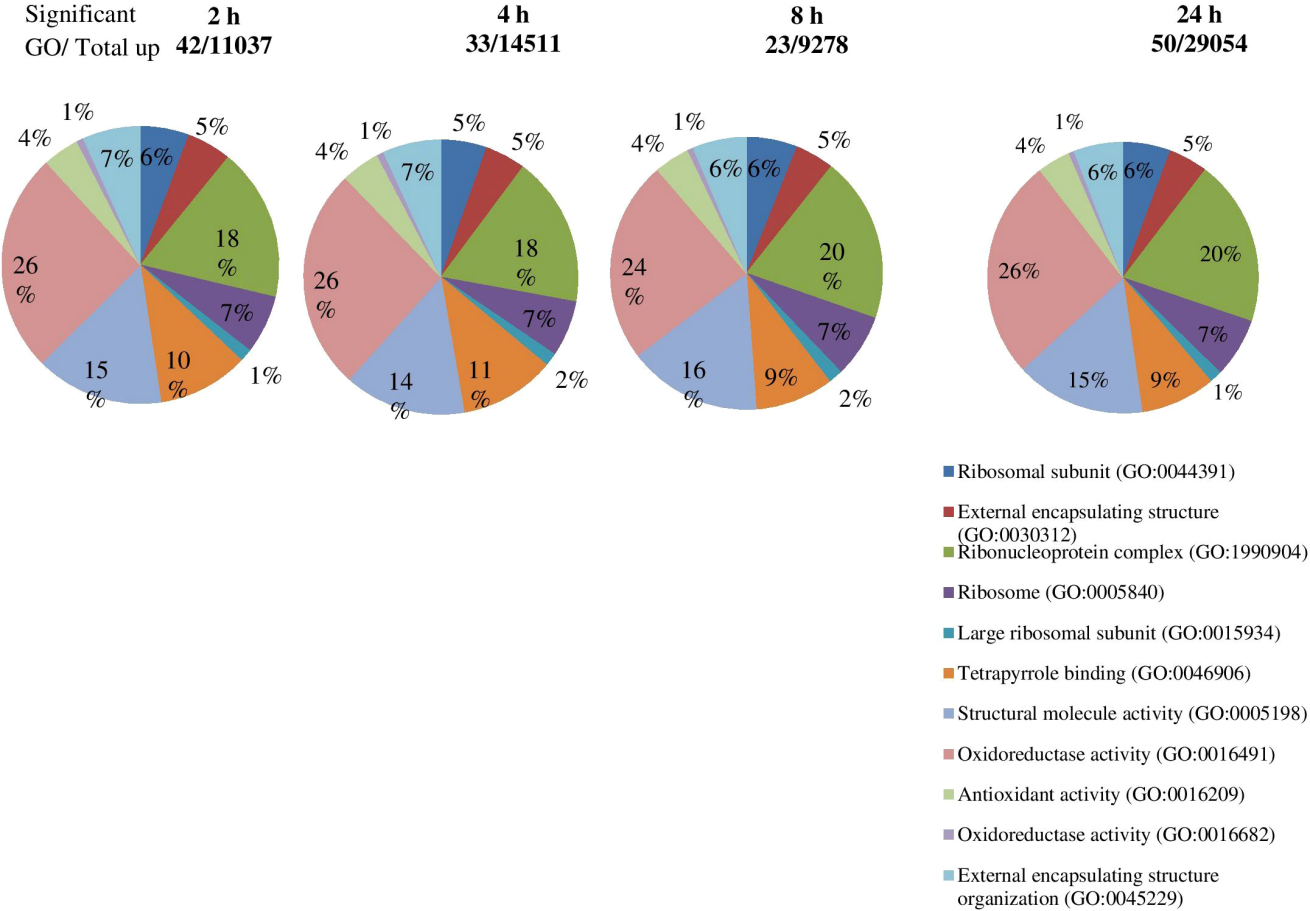
Significant GO enrichment analysis of genes in roots tips of *S. spontaneum* plants in response to drought stress. The number of upregulated genes identified in each comparison of specific time intervals.

The DEGs were significantly enriched to 367 GO IDs, including 149 in a cellular component, 113 in molecular function, and 105 in the biological process. In the cellular component, GO:0016020 (membrane) occupied the first place in the five gene sets, and GO:0044425 (membrane part), GO:0030529 (intracellular ribonucleoprotein complex) also appeared in the top three positions of the five gene sets. In molecular function, GO:0003824 (catalytic activity) occupied the first number in five gene sets, with gene coverage greater than 60%. GO:0016491 (oxidoreductase activity) was significantly enriched in eight gene sets, and the gene coverage in each gene set of about 10%. GO:0005198 structural molecule activity was found in seven gene sets, and the gene coverage was smaller than GO:0016491 (oxidoreductase activity). In the biological process, the top three enriched GO IDs were found in three and mostly in only one gene set. The more coverage rates in one gene set were GO:0044699 (single-organism process), greater than 50%, followed by GO:0044710 (single-organism metabolic process) and GO:0044763 (single-organism cellular function), greater than 30% (**Figure 7**; **Table 4**).

**Table 4:** Significant GO and top three enriched genes in different gene sets.

Gene set	Cellular component	Molecular function	Biological process
CK-vs-T_2_ (42)	GO:0044391,GO:0005840,GO:0030312,GO:0030529,GO:1990904,GO:0031224,GO:0071944,GO:0044425,GO:0005576,GO:0015935,GO:0005618,GO:0030964,GO:0015934,GO:1990204,GO:0016020	GO:0046906,GO:0005198,GO:0016491,GO:0016798,GO:0016757,GO:0016209,GO:0016682,GO:0031127,GO:0004553,GO:0016679,GO:0008417,GO:0016705	GO:0045229,GO:0071554,GO:0071555,GO:0071669,GO:0009698,GO:0009808,GO:0000272,GO:0015698,GO:0048509,GO:0016052,GO:0009826,GO:0006820,GO:0060560,GO:0006811,GO:0009664
	1. GO:0016020 membrane 1859(25.5%) 2. GO:0044425 membrane part 1410(19.34%) 3. GO:0031224 intrinsic component of membrane 1300(17.84%)	1. GO:0016491 oxidoreductase activity 855(9.93%) 2. GO:0005198 structural molecule activity 499(5.8%) 3. GO:0046906 tetrapyrrole binding 353(4.1%)	1. GO:0006811 ion transport 413(5.48%) 2. GO:0045229 external encapsulating structure organization 225(2.99%) 3. GO:0071554 cell wall organization or biogenesis 156(2.07%)
CK-vs-T_4_ (33)	GO:0044391,GO:0005840,GO:0030312,GO:0009521,GO:0030529,GO:1990904,GO:0031224,GO:0034357,GO:0071944,GO:0044425,GO:0015934,GO:0044436,GO:0005576	GO:0046906,GO:0005198,GO:0016757,GO:0016798,GO:0016209,GO:0016491,GO:0016679,GO:0016682,GO:0016709,GO:0031127,GO:0016705,GO:0008417,GO:0003824,GO:0051213,GO:0016758	GO:0045229,GO:0005976,GO:0071554,GO:0071555,GO:0009808
	1. GO:0044425 membrane part 1495(18.81%) 2. GO:0031224 intrinsic component of membrane 1365(17.17%) 3. GO:0030529 intracellular ribonucleoprotein complex 607(7.64%)	1. GO:0003824 catalytic activity 6026(62.67%) 2. GO:0016491 oxidoreductase activity 897(9.33%) 3. GO:0005198 structural molecule activity 494(5.14%)	1. GO:0005976 polysaccharide metabolic process 300(3.59%) 2. GO:0045229 external encapsulating structure organization 236(2.82%) 3. GO:0071554 cell wall organization or biogenesis 166(1.99%)
CK-vs-T_8_ (23)	GO:0030529,GO:1990904,GO:0005840,GO:0044391,GO:0015935,GO:0015934,GO:0071944,GO:0030312,GO:0043228,GO:0043232,GO:0032991	GO:0005198,GO:0046906,GO:0016209,GO:0016491,GO:0016757,GO:0016682	GO:0045229,GO:0071555,GO:0071554,GO:0010467,GO:0000272,GO:0016052
	1. GO:0030529 intracellular ribonucleoprotein complex 774(9.24%) 2. GO:1990904 ribonucleoprotein complex 774(9.24%) 3. GO:0043232 intracellular non-membrane-bounded organelle 641(7.65%)	1. GO:0016491 oxidoreductase activity 943(9.41%) 2. GO:0005198 structural molecule activity 625(6.24%) 3. GO:0016757 transferase activity, transferring glycosyl groups 387(3.86%)	1. GO:0010467 gene expression 981(11.06%) 2. GO:0045229 external encapsulating structure organization 252(2.84%) 3. GO:0071554 cell wall organization or biogenesis 182(2.05%)
CK-vs-T_24_ (50)	GO:0030529,GO:1990904,GO:0005840,GO:0044391,GO:0032991,GO:0030312,GO:0005739,GO:0016020,GO:0043228,GO:0043232,GO:0015935,GO:0044455,GO:0005740,GO:0031966,GO:0071944,GO:0015934,GO:0015630,GO:0044429,GO:0044425,GO:0005856,GO:0005875,GO:0030964,GO:0031224,GO:0005576,GO:0044430,GO:1990204	GO:0005198,GO:0016491,GO:0046906,GO:0003824,GO:0015631,GO:0005215,GO:0016209,GO:0016798,GO:0016903,GO:0003774,GO:0008092,GO:0004180,GO:0016620,GO:0022857,GO:0016614,GO:0016682,GO:0016616,GO:0008483,GO:0016746,GO:0016679	GO:0045229,GO:0010038,GO:0045333,GO:0000272
	1. GO:0016020 membrane 2880(25.9%) 2. GO:0044425 membrane part 2058(18.51%) 3. GO:0031224 intrinsic component of membrane 1839(16.54%)	1. GO:0003824 catalytic activity 8219(62.63%) 2. GO:0016491 oxidoreductase activity 1278(9.74%) 3. GO:0005198 structural molecule activity 755(5.75%)	1. GO:0045229 external encapsulating structure organization 286(2.41%) 2. GO:0010038 response to metal ion 122(1.03%) 3. GO:0045333 cellular respiration 71(0.6%)
T_2_-vs-T_4_ (24)	GO:0005840,GO:0043228,GO:0043232,GO:0044391,GO:0009521,GO:0030529,GO:1990904,GO:0034357,GO:0044436,GO:0015935	–	GO:0090304,GO:0006139,GO:0006725,GO:0046483,GO:0034641,GO:0006974,GO:1901360,GO:0006281,GO:0006807,GO:0000725,GO:0006310,GO:0051321,GO:0033554,GO:1903046
	1. GO:0043232 intracellular non-membrane-bounded organelle 286(8.32%) 2. GO:0030529 intracellular ribonucleoprotein complex 82(8.21%) 3. GO:0043228 non-membrane-bounded organelle 286(8.32%)	–	1. GO:0006807 nitrogen compound metabolic process 945(25.58%) 2. GO:1901360 organic cyclic compound metabolic process 865(23.41%) 3. GO:0006725 cellular aromatic compound metabolic process 856(23.17%)
T_2_-vs-T_8_ (49)	GO:0030529,GO:1990904,GO:0005840,GO:0044391,GO:0032991,GO:0043228,GO:0043232,GO:0015934,GO:0015935,GO:0005576,GO:0044436,GO:0009536,GO:0009579	GO:0005198,GO:0016682,GO:0016679,GO:0004518	GO:0010467,GO:0006725,GO:0046483,GO:0006807,GO:0034641,GO:1901360,GO:0006753,GO:0010605,GO:0016458,GO:0055086,GO:0006139,GO:0009117,GO:0010629,GO:0031047,GO:0009892,GO:0019637,GO:0048519,GO:0016441,GO:0035194,GO:0042126,GO:2001057,GO:0009451,GO:0031050,GO:0043331,GO:0070918,GO:0071359,GO:1901699,GO:0008299,GO:0016246,GO:0030422,GO:0006913,GO:0051169
	1. GO:0032991 macromolecular complex 1005(16.67%) 2. GO:0009536 plastid 711(11.79%) 3. GO:0030529 intracellular ribonucleoprotein complex 569(9.44%)	1. GO:0005198 structural molecule activity 454(6.37%) 2. GO:0004518 nuclease activity 106(1.49%) 3. GO:0016682 oxidoreductase activity, acting on diphenols and related substances as donors, oxygen as acceptor 23 (0.32%)	1. GO:0006807 nitrogen compound metabolic process 1591(25.01%) 2. GO:0006725 cellular aromatic compound metabolic process 1415(22.24%) 3. GO:0034641 cellular nitrogen compound metabolic process 1407(22.12%)
T_2_-vs-T_24_ (41)	GO:0030529,GO:1990904,GO:0005840,GO:0044391,GO:0043228,GO:0043232,GO:0030312,GO:0032991,GO:0016020,GO:0071944,GO:0005576,GO:0015630,GO:0015934,GO:0015935,GO:0005911,GO:0030054,GO:0005737,GO:0005739,GO:0005856,GO:0044444	GO:0016491,GO:0005198,GO:0003824,GO:0008483,GO:0016903,GO:0016620,GO:0046906,GO:0016209,GO:0016627,GO:0016829,GO:0070546,GO:0015925,GO:0016769,GO:0016421,GO:0016885,GO:0051213	GO:0044699,GO:0010038,GO:1901987,GO:1901990,GO:0044710
	1. GO:0016020 membrane 2088(26.11%) 2. GO:0005737 cytoplasm 1786(22.34%) 3. GO:0044444 cytoplasmic part 1743 (21.8%)	1. GO:0003824 catalytic activity 6095(64.06%) 2. GO:0016491 oxidoreductase activity 1011(10.63%) 3. GO:0005198 structural molecule activity 545(5.73%)	1. GO:0044699 single-organism process 4521(52.15%) 2. GO:0044710 single-organism metabolic process 2760(31.83%) 3. GO:0010038 response to metal ion 97(1.12%)
T_4_-vs-T_8_ (25)	GO:0030529,GO:1990904,GO:0005840,GO:0032991,GO:0005576,GO:0044391,GO:0015934,GO:0043228,GO:0043232,GO:0000785,GO:0000109,GO:0071944,GO:0030312,GO:0015935,GO:1990391	GO:0005198,GO:0016491,GO:0016682,GO:0016679,GO:0046906	GO:0072522,GO:0009808,GO:0010038,GO:0006164,GO:0009698
	1. GO:0032991 macromolecular complex 556(18.12%) 2. GO:0030529 intracellular ribonucleoprotein complex 341(11.11%) 3. GO:1990904 ribonucleoprotein complex 341 (11.11%)	1. GO:0016491 oxidoreductase activity 400(10.8%) 2. GO:0005198 structural molecule activity 259(7%) 3. GO:0046906 tetrapyrrole binding 136(3.67%)	1. GO:0010038 response to metal ion 46(1.37%) 2. GO:0072522 purine-containing compound biosynthetic process 45(1.34%) 3. GO:0006164 purine nucleotide biosynthetic process 39 (1.16%)
T_4_-vs-T_24_ (52)	GO:0005856,GO:0016020,GO:0015630,GO:0005875,GO:0044430,GO:0005576,GO:0000793,GO:0043228,GO:0043232,GO:0030312,GO:0005739,GO:0044425,GO:0031224,GO:0005737,GO:0044422,GO:0044446,GO:0071944	GO:0016491,GO:0003824,GO:0016903,GO:0016620,GO:0003774,GO:0015631,GO:0008092,GO:0016209,GO:0008483,GO:0016627,GO:0016829,GO:0015926,GO:0015925,GO:0046906,GO:0016746,GO:0008422,GO:0070546,GO:0051536,GO:0016769,GO:0051540,GO:0005215	GO:0044699,GO:0007017,GO:0044710,GO:0043648,GO:0051052,GO:0034968,GO:0010038,GO:1901987,GO:1901990,GO:0010564,GO:0044711,GO:0015851,GO:0018022,GO:0009064
	1. GO:0016020 membrane 2287(26.35%) 2. GO:0005737 cytoplasm 1927(22.21%) 3. GO:0044425 membrane part 1619(18.66%)	1. GO:0003824 catalytic activity 6504(64.05%) 2. GO:0016491 oxidoreductase activity 1014(9.99%) 3. GO:0005215 transporter activity 557(5.49%)	1. GO:0044699 single-organism process 4894(52.69%) 2. GO:0044710 single-organism metabolic process 2958(31.84%) 3. GO:0044711 single-organism biosynthetic process 726(7.82%)
T_8_-vs-T_24_ (28)	GO:0016020,GO:0005576,GO:0044430,GO:0015630,GO:0005875,GO:0005856,GO:0030312,GO:0005739,GO:0044425	GO:0003824,GO:0016491,GO:0016903,GO:0008483,GO:0016769,GO:0016620,GO:0015631,GO:0003774,GO:0008092,GO:0016829,GO:0016209,GO:0016746,GO:0051536,GO:0016682	GO:0044699,GO:0044710,GO:0009698,GO:0044763,GO:0043648
	1. GO:0016020 membrane 2227(26.31%) 2. GO:0044425 membrane part 1570(18.55%) 3. GO:0005856 cytoskeleton 163(1.93%)	1. GO:0003824 catalytic activity 6499(65.04%) 2. GO:0016491 oxidoreductase activity 976(9.77%) 3. GO:0016829 lyase activity 244(2.44%)	1. GO:0044699 single-organism process 4796(53.15%) 2. GO:0044763 single-organism cellular process 3291(36.47%) 3. GO:0044710 single-organism metabolic process 909(32.24%)

### KEGG analysis of differentially expressed genes

**Figure 8:**
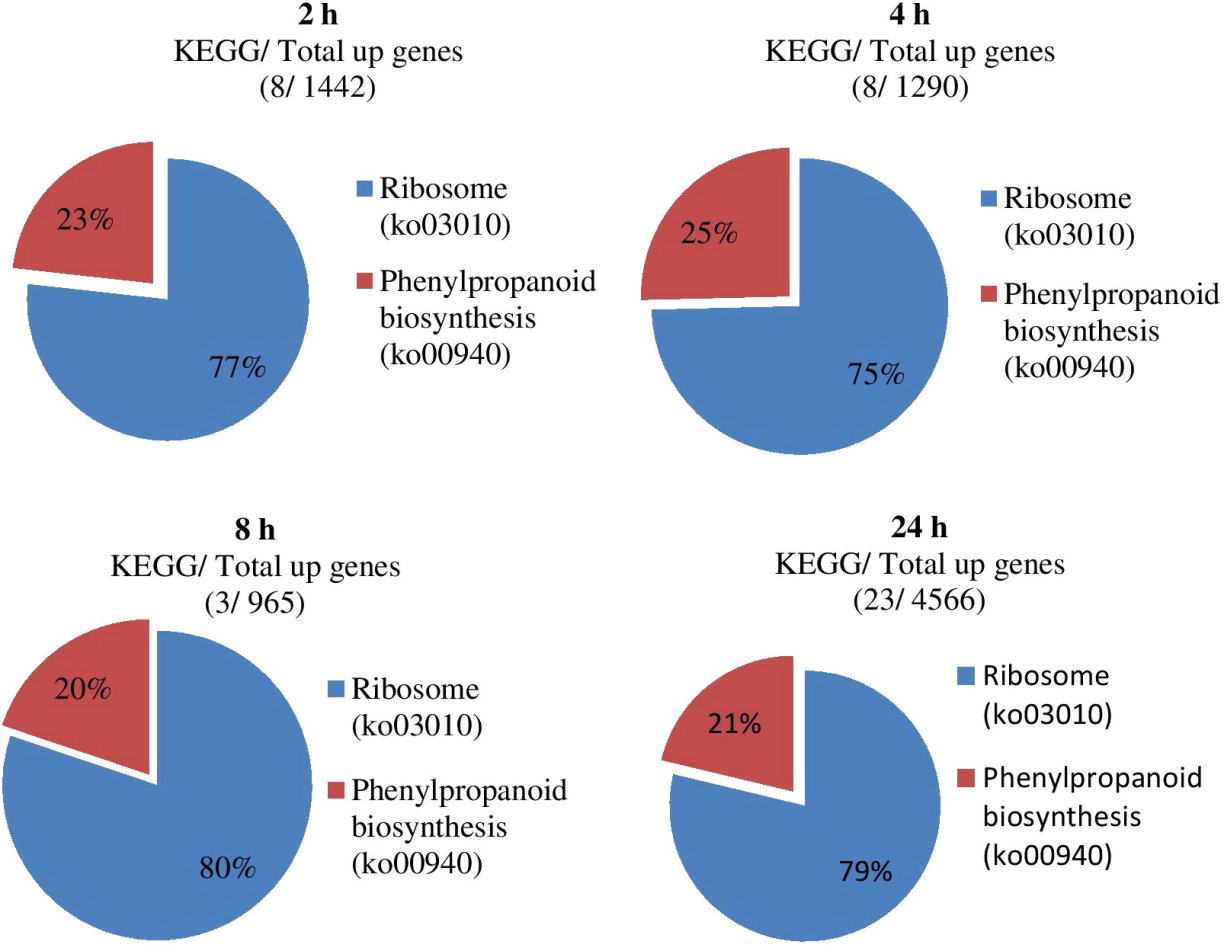
Impact of significant KEGG enrichment at different gene sets during stress conditions.

The KEGG analysis results of all DEGs showed that the fifty-five significant pathways (42.31%) were enriched, and a total of 9056 genes distributed in ten gene sets (**Figure 8**; **Table 5**). ko03010 (Ribosome) was significantly enriched in eight gene sets and the pathway with the most significant number of genes in each gene set. ko00940 (Phenylpropanoid biosynthesis) and ko00190 (Oxidative phosphorylation) were found in seven and six gene sets, respectively. It can also be drawn from **Table 5** that the three gene sets were significantly enriched, i.e., CK-vs-T_24_ (6024), T_4_-vs-T_24_ (4862) and T_2_-vs-T_24_ (4750). T4-vs-T24 (33 pathways) and low T2-vs-T8 (1 pathway) were enriched considerably.

**Table 5:** Results of significant KEGG enrichment in different gene sets.

Gene set	Significant pathway/total pathway	DEGs/All genes with pathway annotation	Significant Pathway ID (Pathway, DEGs genes, Q value)
CK-vs-T_2_	8/128	3405/9056	ko03010(Ribosome, 594 (17.44%), 0.000000) ko00940(Phenylpropanoid biosynthesis,180 (5.29%), 0.000000) ko00480(Glutathione metabolism,123 (3.61%), 0.000030) ko04075(Plant hormone signal transduction,131 (3.85%), 0.019840) ko00941(Flavonoid biosynthesis,34 (1%), 0.022968) ko00052(Galactose metabolism),60 (1.76%), 0.035416) ko00500(Starch and sucrose metabolism,125 (3.67%), 0.047087) ko00190(Oxidative phosphorylation,195 (5.73%), 0.048035)
CK-vs-T_4_	8/127	3652/9056	ko00940(Phenylpropanoid biosynthesis,201 (5.5%), 0.000000) ko03010(Ribosome,591 (16.18%), 0.000000) ko00480(Glutathione metabolism,141 (3.86%), 0.000000) ko04075(Plant hormone signal transduction,148 (4.05%), 0.000216) ko00500(Starch and sucrose metabolism,144 (3.94%), 0.000338) ko00196(Photosynthesis - antenna proteins,17 (0.47%), 0.009984) ko00941(Flavonoid biosynthesis, 36 (0.99%), 0.011822) ko0051(Other types of O-glycan biosynthesis, 12 (0.33%), 0.031494)
CK-vs-T_8_	3/128	4330/9056	ko03010(Ribosome,800(18.48%),0.000000) ko00940(Phenylpropanoid biosynthesis,19 (4.57%),0.000000) ko00480(Glutathione metabolism,146(3.37%),0.000058)
CK-vs-T_24_	23/130	6024/9056	ko03010(Ribosome,944(15.67%),0.000000) ko00940(Phenylpropanoid biosynthesis,255(4.23%),0.000000) ko00190(Oxidative phosphorylation,352(5.84%),0.000000) ko01200(Carbon metabolism,493(8.18%),0.000003) ko00280(Valine, leucine and isoleucine degradation,122(2.03%),0.000271) ko00640(Propanoate metabolism,80(1.33%),0.000796) ko04145(Phagosome,151(2.51%),0.001541) ko00620(Pyruvate metabolism,155(2.57%),0.002733) ko00071(Fatty acid degradation,96(1.59%),0.006303) ko01230(Biosynthesis of amino acids,365(6.06%),0.006303) ko00010(Glycolysis/Gluconeogenesis,221(3.67%),0.007751) ko00330(Arginine and proline metabolism,88 1.46%),0.007751) ko00250(Alanine, aspartate and glutamate metabolism,123(2.04%),0.011469) ko00020(Citrate cycle (TCA cycle),135(2.24%),0.012199) ko00350(Tyrosine metabolism,79(1.31%),0.012510) ko00051(Fructose and mannose metabolism,100(1.66%),0.012602) ko00030(Pentose phosphate pathway,100(1.66%),0.012602) ko00710(Carbon fixation in photosynthetic organisms,138(2.29%),0.013118) ko00950(Isoquinoline alkaloid biosynthesis,41(0.68%),0.018554) ko00270(Cysteine and methionine metabolism,154(2.56%),0.020575) ko00520(Amino sugar and nucleotide sugar metabolism,175(2.91%),0.029370) ko01212(Fatty acid metabolism,139(2.31%),0.038864) ko00380(Tryptophan metabolism,60(1%),0.041102)
T_2_-vs-T_4_	6/126	1820/9056	ko03010(Ribosome,283(15.55%),0.000088) ko00190(Oxidative phosphorylation,125(6.87%),0.001171) ko00630(Oxidative phosphorylation,125(6.87%)0.001171) ko03440(Homologous recombination,38(2.09%),0.022462) ko00910(Nitrogen metabolism,24(1.32%),0.034340) ko00196(Photosynthesis - antenna proteins,11(0.6%),0.034340)
T_2_-vs-T_8_	1/127	3225/9056	ko03010(Ribosome,582(18.05%),0.000000)
T_2_-vs-T_24_	28/129	4750/9056	ko030109(Ribosome,729(15.35%),0.000000) ko01200(Carbon metabolism,419(8.82%),0.000000) ko00250(Alanine, aspartate and glutamate metabolism,121(2.55%),0.000000) ko00940(Phenylpropanoid biosynthesis1,99 (4.19%),0.000005) ko00280(Valine, leucine and isoleucine degradation,108(2.27%),0.000005) ko00710(Carbon fixation in photosynthetic organisms,124(2.61%),0.000094) ko00190(Oxidative phosphorylation,278(5.85%),0.000112) ko00640(Propanoate metabolism,70(1.47%),0.000145) ko01212(Fatty acid metabolism,125(2.63%),0.000256) ko00051(Fructose and mannose metabolism,89(1.87%),0.000452) ko00100(Steroid biosynthesis,53(1.12%),0.000596) ko00010(Glycolysis/Gluconeogenesis,186(3.92%),0.000989) ko00030(Pentose phosphate pathway,87(1.83%),0.001539) ko00330(Arginine and proline metabolism,76(1.6%),0.001539) ko00410(beta-Alanine metabolism,63(1.33%),0.004172) ko01040(Biosynthesis of unsaturated fatty acids,66(1.39%)0.004172) ko00071(Fatty acid degradation,80(1.68%)0.005390) ko00360(Phenylalanine metabolism,61(1.28%)0.006538) ko00520(Amino sugar and nucleotide sugar metabolism,147(3.09%),0.0066) ko01230(Biosynthesis of amino acids,295(6.21%),0.006617) ko00950(Isoquinoline alkaloid biosynthesis,36(0.76%),0.006875) ko00480(Glutathione metabolism,144(3.03%),0.007974) ko00350(Tyrosine metabolism,66(1.39%),0.010554) ko00020(Citrate cycle (TCA cycle),110(2.32%),0.017396) ko04145(Phagosome,119(2.51%),0.018060) ko00630(Glyoxylate and dicarboxylate metabolism,119(2.51%),0.028353) ko00061(Fatty acid biosynthesis,63(1.33%),0.028353) ko00220(Arginine biosynthesis,50(1.05%),0.039841)
T_4_-vs-T_8_	11/127	2073/9056	ko03010(Ribosome,369(17.8%),0.000000) ko01200(Carbon metabolism,204(9.84%),0.000012) ko00190(Oxidative phosphorylation,142(6.85%),0.000175) ko00020(Citrate cycle (TCA cycle),66(3.18%),0.000188) ko00010(Glycolysis/Gluconeogenesis,98(4.73%),0.000534) ko00250(Alanine, aspartate and glutamate metabolism,55 (2.65%),0.008438) ko00340(Histidine metabolism1,6(0.77%),0.019835) ko00565(Ether lipid metabolism,21(1.01%),0.031616) ko00630(Glyoxylate and dicarboxylate metabolism,61(2.94%),0.042868) ko00052(Galactose metabolism,40(1.93%),0.042868) ko01230(Biosynthesis of amino acids,139(6.71%),0.047970)
T_4_-vs-T_24_	33/127	4862/9056	ko01200(Carbon metabolism,430(8.84%),0.000000) ko00250(Alanine, aspartate and glutamate metabolism,123(2.53%),0.000000) ko00071(Fatty acid degradation,93(1.91%),0.000001) ko01212(Fatty acid metabolism,134(2.76%),0.000003) ko00280(Valine, leucine and isoleucine degradation,110(2.26%),0.000004) ko00330(Arginine and proline metabolism,83(1.71%),0.000020) ko00640(Propanoate metabolism,72 (1.48%),0.000061) ko00010(Glycolysis/Gluconeogenesis, 195 (4.01%),0.000084) ko00940(Phenylpropanoid biosynthesis, 196 (4.03%),0.000116) ko00620(Pyruvate metabolism, 135 (2.78%),0.000243) ko00061(Fatty acid biosynthesis,71 (1.46%),0.000380) ko00650(Butanoate metabolism,51 (1.05%),0.000642) ko00710(Carbon fixation in photosynthetic organisms,122 (2.51%),0.000704) ko00020(Citrate cycle (TCA cycle),118 (2.43%),0.001169) ko01230(Biosynthesis of amino acids,307 (6.31%),0.001169) ko00100(Steroid biosynthesis,52 (1.07%),0.002437) ko00520(Amino sugar and nucleotide sugar metabolism,152 (3.13%),0.002646) ko01040(Biosynthesis of unsaturated fatty acids,67 (1.38%),0.003973) ko04145(Phagosome,124(2.55%),0.006474) ko00400(Phenylalanine, tyrosine and tryptophan biosynthesis,53(1.09%),0.007471) ko00564(Glycerophospholipid metabolism,119(2.45%),0.009043) ko00565(Ether lipid metabolism,37(0.76%),0.014621) ko00051(Fructose and mannose metabolism,84(1.73%),0.015415) ko00270(Cysteine and methionine metabolism,129(2.65%),0.017672) ko00592(alpha-Linolenic acid metabolism,64(1.32%),0.022818) ko00903(Limonene and pinene degradation,11(0.23%),0.031665) ko00600(Sphingolipid metabolism,47(0.97%),0.031781) ko00040(Pentose and glucuronate interconversions,53(1.09%),0.032424) ko01210(2-Oxocarboxylic acid metabolism,91(1.87%),0.032487) ko00380(Tryptophan metabolism,51 (1.05%),0.033757) ko00410(beta-Alanine metabolism,60 (1.23%),0.033757) ko00561(Glycerolipid metabolism,72 (1.48%),0.042839) ko00430(Taurine and hypotaurine metabolism,22 (0.45%),0.046042)
T_8_-vs-T_24_	28/127	4662/9056	ko00071(Fatty acid degradation,94(2.02%),0.000000) ko00280(Valine, leucine and isoleucine degradation,112(2.4%),0.000000) ko01200(Carbon metabolism,408(8.75%),0.000000) ko00250(Alanine, aspartate and glutamate metabolism,115(2.47%),0.000001) ko01212(Fatty acid metabolism,130 (2.79%),0.000003) ko00640(Propanoate metabolism,71 (1.52%),0.000028) ko00650(Butanoate metabolism,52 (1.12%),0.000073) ko00010(Glycolysis/Gluconeogenesis,187 (4.01%),0.000214) ko00061(Fatty acid biosynthesis,69 (1.48%),0.000479) ko00100(S teroid biosynthesis,52 (1.12%),0.000958) ko00620(Pyruvate metabolism,128 (2.75%),0.001224) ko00940(Phenylpropanoid biosynthesis,184 (3.95%),0.001491) ko00400(Phenylalanine, tyrosine and tryptophan biosynthesis,53 (1.14%),0.003082) ko00790(Folate biosynthesis,20 (0.43%),0.003608) ko01230(Biosynthesis of amino acids,292 (6.26%),0.004906) ko01220(Degradation of aromatic compounds,18(0.39%),0.009123) ko00020(Citrate cycle (TCA cycle),110(2.36%),0.010722) ko00520(Amino sugar and nucleotide sugar metabolism,143(3.07%),0.014530) ko00710(Carbon fixation in photosynthetic organisms,112(2.4%),0.014530) ko01040(Biosynthesis of unsaturated fatty acids,63(1.35%),0.014791) ko00565(Ether lipid metabolism,36(0.77%),0.014894) ko00330(Arginine and proline metabolism,71(1.52%),0.014992) ko00270(Cysteine and methionine metabolism,124(2.66%) 0.023659) ko00410(beta-Alanine metabolism59(1.27%),0.024742) ko00564(Glycerophospholipid metabolism,112(2.4%),0.030677) ko00531(Glycosaminoglycan degradation,18(0.39%),0.038118) ko00190(Oxidative phosphorylation,253(5.43%),0.046100) ko00310(Lysine degradation,49(1.05%),0.047302)

### Trend analysis of expressed genes

All expressed genes were analyzed by Short Time-series Expression Miner software and corrected by FDR. With Q value ≤ 0.05 as the threshold, a total of 53683 trend genes, 20 trend lines, and 6 significant trend lines were observed (**Figure S8**). In trend profile 18, a total of 9,548 genes, the gene expression trend showed a significant upward trend within 0-4 h in response to PEG-6000 (25%) stressed condition. The expression level remained similar between 4 and 8 h and decreased slightly after 8 h. However, the CK was still up-regulated (**Figure S9A**). In profile 19, a total of 8,876 genes were observed and showed a continuous upward trend of gene expression from 0 - 24 h during drought stress condition (**Figure S9B**). In trend profile 0, a total of 7,207 genes were found, and the trend was different to profile 19. The gene expression showed a continuous downregulated pattern from 0 - 24 h (**Figure S9C**). A total of 5,195 genes were found in profile 5; the gene expression pattern showed a significant decrease from 0-2 h followed by an increase in 2-4 h, it remained stable at 4-8 h, and decreased again after 8 h (**Figure S9D**). In profile16 (4140 genes) was similar to profile18 with significantly increasing trend within 0-4 h, slight reduction between 4 and 8 h, and downregulated after 8 h (**Figure S9E**). A total of 2,477 genes were observed in profile 2 and similar to profile 5, with significant decrease from 0-2 h, relatively stable in the following 2-4 h, increase in 4-8 h and decline again after 8 h (**Figure S9F**).

### Trend genes GO enrichment analysis

**Figure 9:**
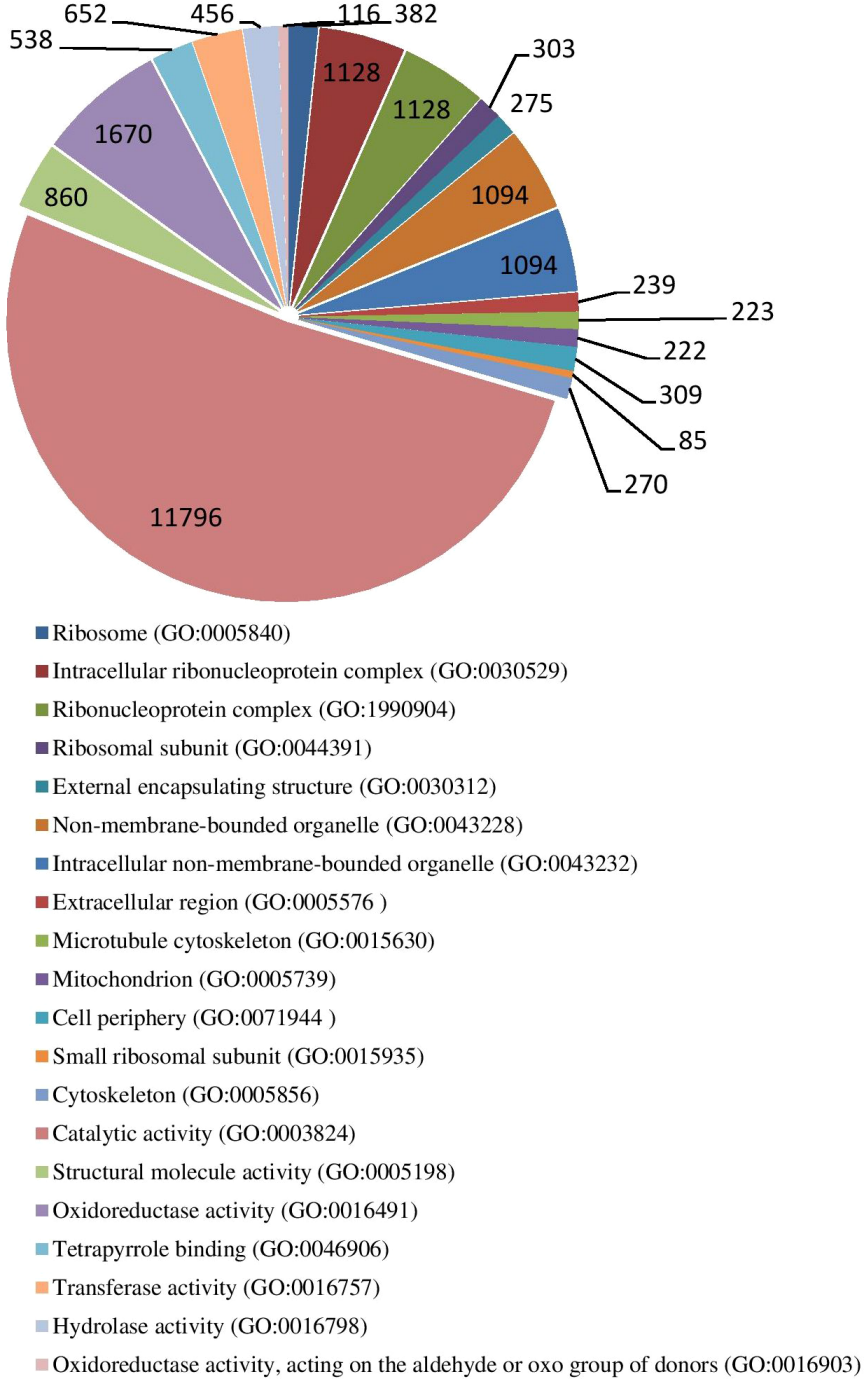
Gene ontology numbers indicated in significant GO IDs in all trends.

**Figure 10:**
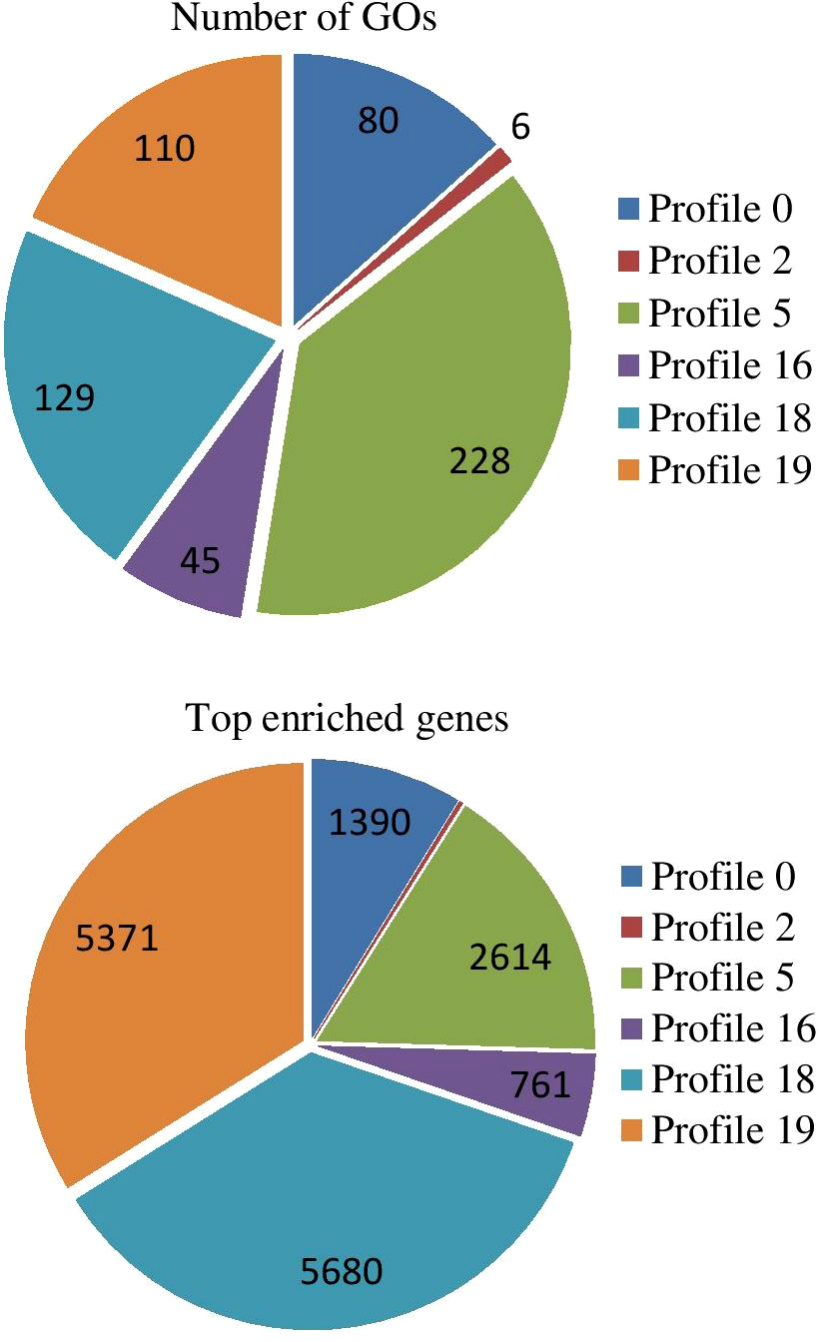
Significant GO enrichment analyses of top enriched genes in different trends.

The results of GO enrichment analysis of all trend genes showed a total of 2,986 GO IDs, with 20 significantly enriched, including 13 in a cellular component, 7 in molecular functions, and 0 in the biological process (**Figure 9**; **Table 6**). The GO enrichment analysis of 6 significant trends showed the cellular component, profiles 0 and 19 were enriched with the most GO IDs, both of which were 22, while profile 2 was 0. GO:0044425 (membrane part) and GO:0031224 (an intrinsic membrane component) were enriched in 3 trend sets. In molecular function, profile 18 was found to be enriched to the most GO IDs (32), and GO:0016491 (oxidoreductase activity) and GO:0005198 (structural molecule activity) were found in 2 trend sets. GO: 0005488 (binding) and GO: 0003824 (catalytic activity) were found in profiles 18 and 19 with different genes. In biological process, profiles 5, 18, and 19 were enriched with more GO IDs, i.e., 194, 87 and 73, respectively. GO:0008152 (metabolic process) and GO:0071704 (organic substance metabolic process) have a large number of genes, which are enriched in profiles 18 and 19 (**Figure 10**; **Table 7**).

**Table 6:** Significant GO ID in all trends.

GO Enrichment	GO ID (description, gene ratio, Q value)
Cellular Component	GO:0005840 ribosome 382(2.4%) 0.000009 GO:0030529 intracellular ribonucleoprotein complex 1128(7.09%) 0.000033 GO:1990904 ribonucleoprotein complex 1128(7.09%) 0.000033 GO:0044391 ribosomal subunit 303(1.91%) 0.000051 GO:0030312 external encapsulating structure 275(1.73%) 0.000701 GO:0043228 non-membrane-bounded organelle 1094(6.88%) 0.001340 GO:0043232 intracellular non-membrane-bounded organelle 1094(6.88%) 0.001340 GO:0005576 extracellular region 239(1.5%) 0.001340 GO:0015630 microtubule cytoskeleton 223(1.4%) 0.001340 GO:0005739 mitochondrion 222(1.4%) 0.006077 GO:0071944 cell periphery 309(1.94%) 0.006077 GO:0015935 small ribosomal subunit 85(0.53%) 0.020955 GO:0005856 cytoskeleton 270(1.7%) 0.020955
Molecular Function	GO:0003824 catalytic activity 11796(62.55%) 0.000000 GO:0005198 structural molecule activity 860(4.56%) 0.000000 GO:0016491 oxidoreductase activity 1670(8.85%) 0.000000 GO:0046906 tetrapyrrole binding 538(2.85%) 0.000023 GO:0016757 transferase activity, transferring glycosyl groups 652(3.46%) 0.000198 GO:0016798 hydrolase activity, acting on glycosyl bonds 456(2.42%) 0.008469 GO:0016903 oxidoreductase activity, acting on the aldehyde or oxo group of donors 116(0.62%) 0.045440
Biological Process	–

**Table 7:** Significant GO Enrichment in six significant trends.

Trend set	Significant GO N. and top three enriched genes (description, gene ratio, qvalue)		
	Cellular Component	Molecular Function	Biological Process
Profile 0	22	16	42
	1.GO:0044425 membrane part 645(20.02%) 0.000684 2.GO:0032991 macromolecular complex 615(19.09%) 0.000000 3.GO:0031224 intrinsic component of membrane 592(18.38%)0.000131	1.GO:0016491 oxidoreductase activity 353(9.79%) 0.015005 2.GO:0005198 structural molecule activity 343(9.52%) 0.000000 3.GO:0046906 tetrapyrrole binding 208(5.77%) 0.000000	1.GO:0010467 gene expression 392(12.07%) 0.000939 2.GO:0045229 external encapsulating structure organization 139(4.28%) 0.000000 3.GO:0005976 polysaccharide metabolic process 123(3.79%) 0.02604
Profile 2	0	3	3
	–	1.GO:0034062 RNA polymerase activity 16(1.34%) 0.013665 2.GO:0016682 oxidoreductase activity, acting on diphenols and related substances as donors, oxygen as acceptor 9(0.75%) 0.013665 3.GO:0016679 oxidoreductase activity, acting on diphenols and related substances as donors 9(0.75%) 0.013665	1.GO:0009698 phenylpropanoid metabolic process 15(1.46%) 0.047329 2.GO:0009808lignin metabolic process 7(0.68%) 0.047329 3.GO:0006796 phosphate-containing compound metabolic process 214(20.84%) 0.047329
Profile 5	17	17	194
	1.GO:0044425 membrane part 462(20.5%) 0.006976 2.GO:0031224 intrinsic component of membrane 424(18.81%) 0.003133 3.GO:0044422 organelle part 415(18.41%) 0.011410	1.GO:0097159 organic cyclic compound binding 1251(51.48%) 0.047059 2.GO:1901363 heterocyclic compound binding 1148(47.24%) 0.031124 3.GO:0036094 small molecule binding 785(32.3%) 0.000318	1.GO:0044763 single-organism cellular process 901(40.44%)0.000002 2.GO:0044260 cellular macromolecule metabolic process 943(42.32%) 0.008138 3.GO:0019538 protein metabolic process 603(27.06%) 0.001916
Profile 16	2	9	34
	1.GO:0031224 intrinsic component of membrane 287(20.08%) 0.000948 2.GO:0044425 membrane part 303(21.2%) 0.015800	1.GO:0005215 transporter activity 108(6.75%) 0.037856 2.GO:0022857 transmembrane transporter activity 103(6.44%) 0.015783 3.GO:0016757 transferase activity, transferring glycosyl groups 77(4.82%) 0.020836	1.GO:0065007 biological regulation 366(25.81%) 0.009113 2.GO:0050789 regulation of biological process 333(23.48%) 0.033428 3.GO:0044255 cellular lipid metabolic process 92(6.49%) 0.03773
Profile 18	10	32	87
	1.GO:0005623cell1924(92.72%)0.008543 2.GO:0044464 cell part 1924(92.72%) 0.008543 3.GO:0005622 intracellular 1894(91.28%) 0.008543	1.GO:0005488 binding1926(72.65%) 0.000001 2.GO:0097159 organic cyclic compoundbinding 1454(54.85%) 0.000000 3.GO:1901363 heterocycliccompound binding 1246(47%) 0.021553	1.GO:0008152 metabolic process 1830(81.59%) 0.000042 2.GO:0009987 cellular process 1730(77.13%) 0.000000 3.GO:0071704 organic substance metabolic process 1510 (67.32%) 0.000000
Profile 19	22	15	73
	1.GO:0005737 cytoplasm 622(30.88%) 0.000000 2.GO:0044444 cytoplasmic part 603(29.94%) 0.000000 3.GO:0032991 macromolecular complex 384(19.07%) 0.000000	1.GO:0003824 catalytic activity 1616(64.28%) 0.037703 2.GO:0016491 oxidoreductase activity 272(10.82%) 0.000266 3.GO:0005198 structuralmolecule activity 172(6.84%) 0.000000	1.GO:0008152 metabolic process 1925(81.16%) 0.000615 2.GO:0071704 organic substance metabolic process 1568(66.1%) 0.000047 3.GO:0006807 nitrogen compound metabolic process 623(26.26%) 0.002907

### Trend genes KEGG enrichment analysis

**Figure 11:**
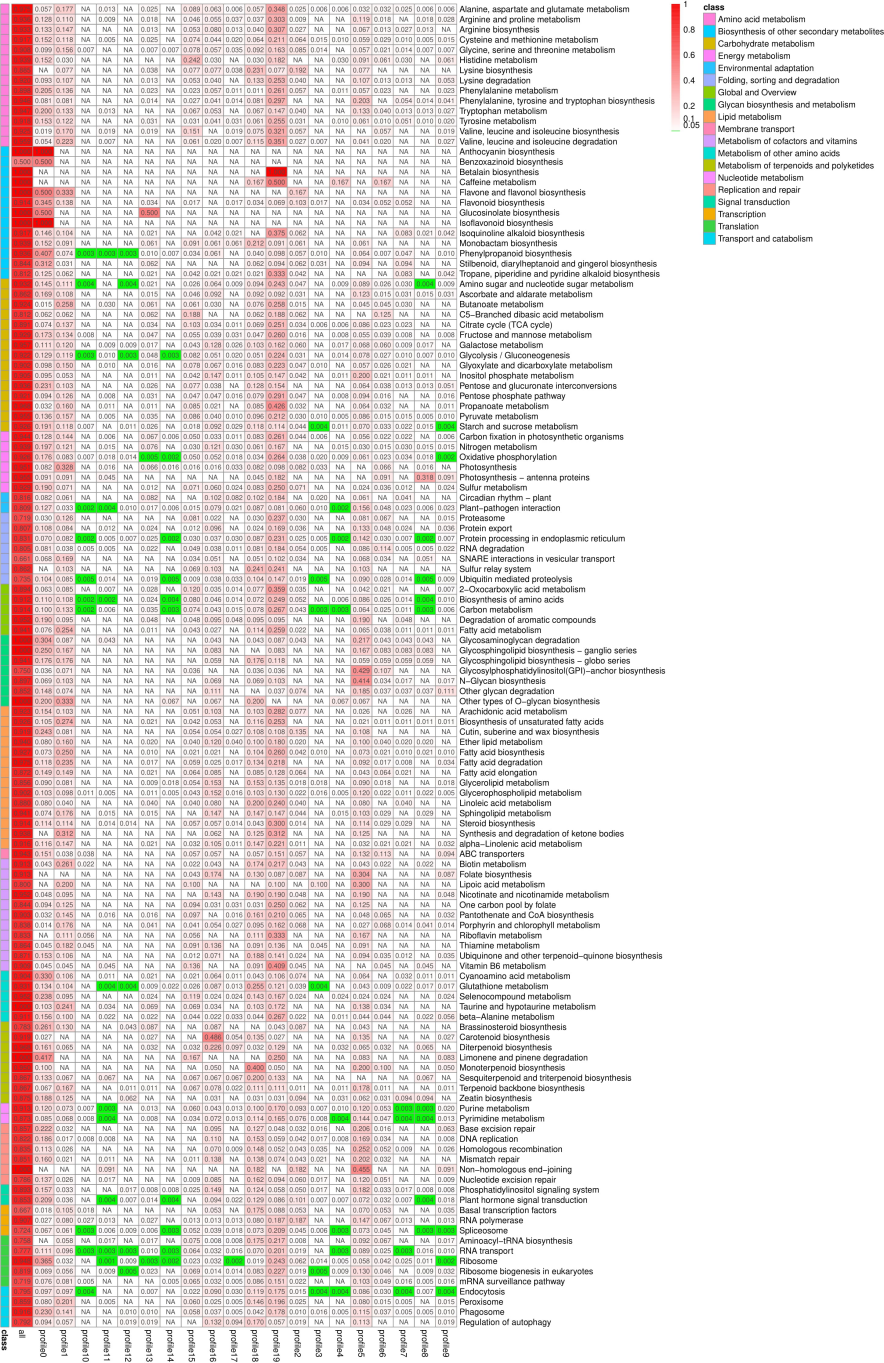
Pathway enrichment analysis rich factor heat map. NA, Not applicable.

**Figure 12:**
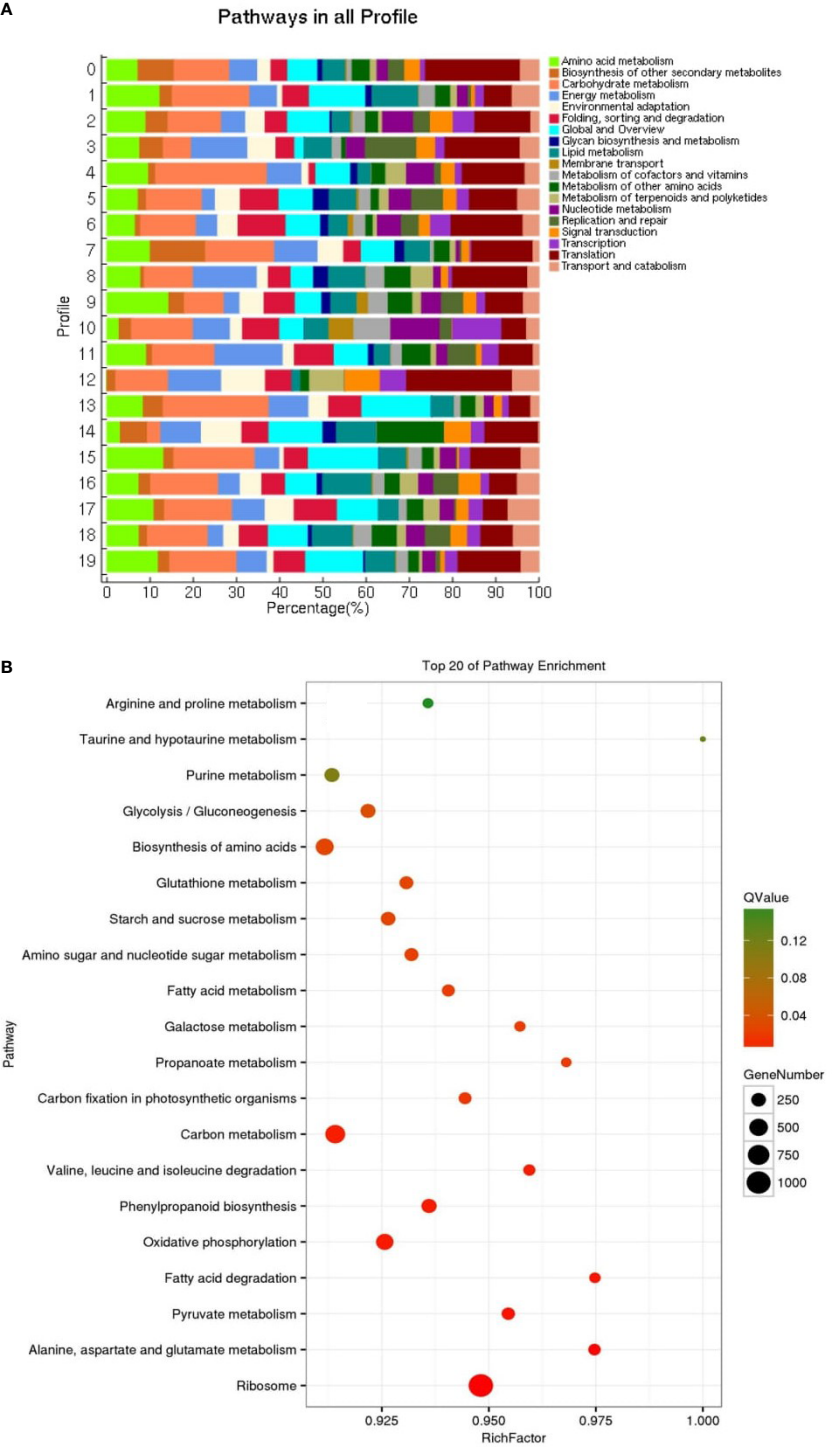
Distribution pathway **(A)** in all profiles and pathway enrichment **(B)** of all genes (top 20).

KEGG enrichment analysis was performed on all trend genes, and a total of 19 KEGG B class pathways and 130 KEGG pathways were observed (**Figure 11**). Pathway-related genes account for more percentage in the distribution of each trend gene set, with carbon metabolism (25.14%), translation (21.35%), and global and overview accounting for 17.36% (**Figure 12A**). More genes were significantly enriched in the pathways such as the ribosome (ko030101042; 13.19%), carbon metabolism (ko01200596; 7.55%), biosynthesis of amino acids (ko01230454; 5.75%), as shown in **Figure 12B**. KEGG analysis of 6 significant trends showed 42 effective pathways were enriched, and 5,630 DEGs were distributed in 6 trend sets (**Table 8**). The most important pathways and DEGs were enriched in profile 19. Ribosome (ko03010) pathway was found most enriched genes in profiles 0, 2 and 19.

**Table 8:** Significant KEGG ID in six significant trends.

Trend set	Significant pathway/Total pathway	DEGs/All genes with pathway annotation	Significant ko ID (Pathway, DEGs N., Q value)
Profile0	4/120	1394/9056	ko03010 (Ribosome, 393 (28.19%), 0.000000) ko00940(Phenylpropanoid biosynthesis,121(8.68%),0.000000) ko00460(Cyanoamino acid metabolism,31(2.22%),0.000627) ko00941(Flavonoid biosynthesis,20(1.43%)0.007527)
Profile2	4/99	408/9056	ko03020(RNA polymerase,14(3.43%),0.000509) ko04075(Plant hormone signal transduction,28(6.86%),0.002605) ko00230(Purine metabolism,28(6.86%),0.006498) ko03010(Ribosome,70(17.16%),0.036715)
Profile5	12/115	875/9056	ko00510(N-Glycan biosynthesis,24(2.74%),0.000000) ko03440(Homologous recombination,29(3.31%),0.000054) ko00563((Glycosylphosphatidylinositol(GPI)-anchor biosynthesis,12(1.37%),0.000166)) ko04626(Plant-pathogen interaction,76(8.69%),0.000266) ko03430(Mismatch repair,19(2.17%),0.029908) ko00562(Inositol phosphate metabolism,19(2.17%),0.029908) ko04141(Protein processing in endoplasmic reticulum,57(6.51%),0.029908) ko03410(Base excision repair,14(1.6%),0.029908) ko03450(Non-homologous end-joining, 5 (0.57%) 0.029908) ko04070(Phosphatidylinositol signaling system,22 (2.51%), 0.029943) ko00400(Phenylalanine, tyrosine and tryptophan biosynthesis,15 (1.71%), 0.043948) ko00790(Folate biosynthesis,7(0.8%),0.045174)
Profile16	6/111	530/9056	ko00906(Carotenoid biosynthesis,18(3.4%),0.000000) ko00564(Glycerophospholipid metabolism,28(5.28%),0.000151) ko04070(Phosphatidylinositol signaling system,18(3.4%),0.006359) ko00561(Glycerolipid metabolism,17(3.21%),0.006359) ko00562(Inositol phosphate metabolism,14(2.64%),0.026252) ko00904(Diterpenoid biosynthesis,7(1.32%),0.032094)
Profile18	2/118	799/9056	ko00480(Glutathione metabolism,59(7.38%),0.000000) ko00902(Monoterpenoid biosynthesis,8(1%),0.010067)
Profile19	14/123	1624/9056	ko01200(Carbon metabolism,174(10.71%),0.000000) ko03010(Ribosome,268(16.5%),0.000000) ko00640(Propanoate metabolism,40(2.46%),0.000001) ko00280(Valine, leucine and isoleucine degradation,53(3.26%),0.000004) ko01210(2-Oxocarboxylic acid metabolism,51(3.14%)0.000005) ko00250(A lanine, aspartate and glutamate metabolism,52(3.2%),0.000069) ko00190(Oxidative phosphorylation,117(7.2%),0.000069) ko01230(Biosynthesis of amino acids,124(7.64%),0.000588) ko00950(Isoquinoline alkaloid biosynthesis,18(1.11%),0.013108) ko00330(Arginine and proline metabolism,33(2.03%),0.013108) ko00030(Pentose phosphate pathway37(2.28%),0.013108) ko01212(Fatty acid metabolism,50(3.08%),0.013108) ko00710(Carbon fixation in photosynthetic organisms,47(2.89%),0.035011) ko00100(Steroid biosynthesis,22(1.35%),0.037385)

### Gene validation

**Figure 13:**
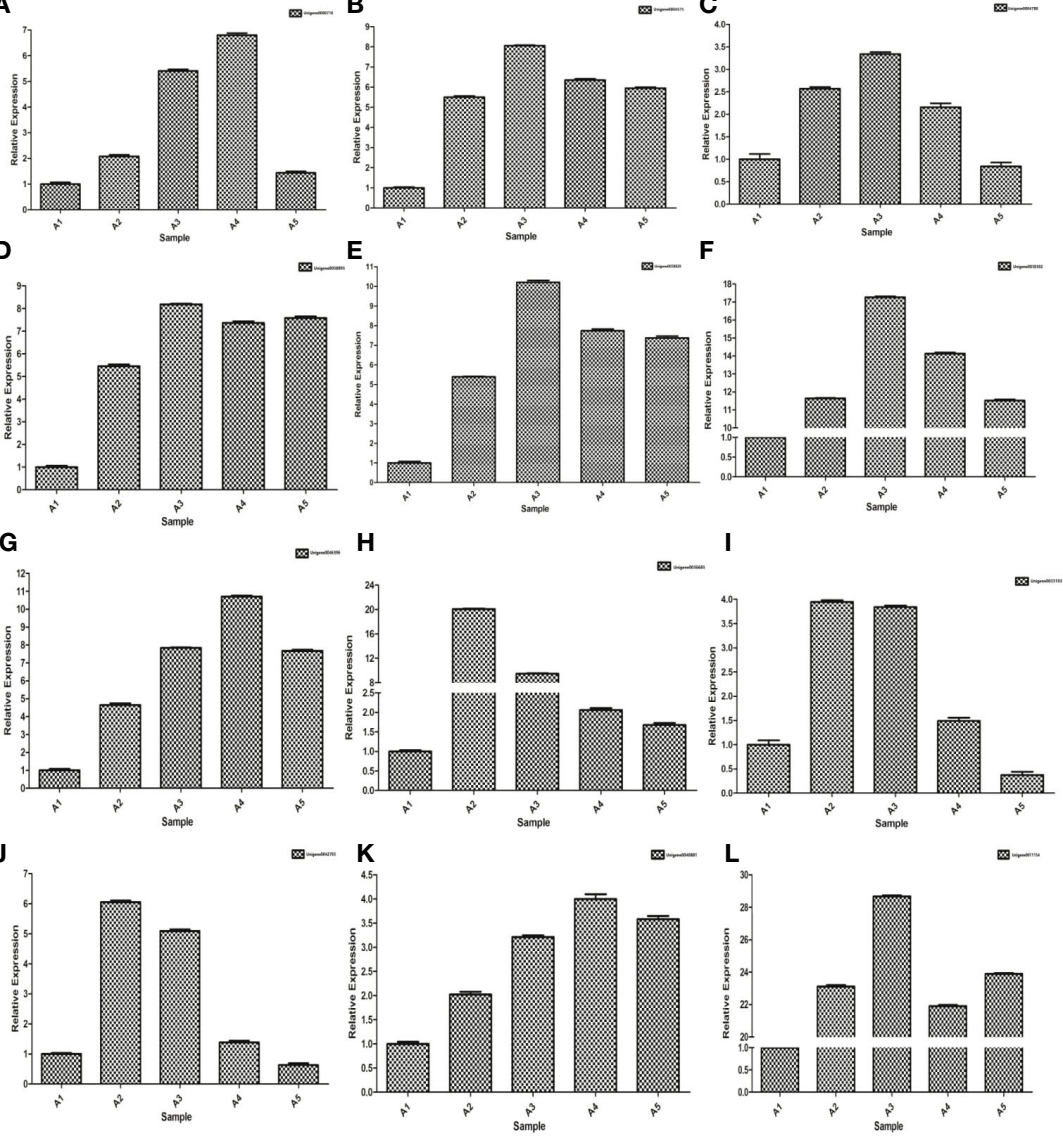
qRT-PCR expression validations of 12 genes. **(A)** Unigene0000718, **(B)** Unigene0004575, **(C)** Unigene0004788, **(D)** Unigene0011154, **(E)** Unigene0033103, **(F)** Unigene0036685, **(G)** Unigene0038302, **(H)** Unigene0038820, **(I)** Unigene004038895, **(J)** Unigene0040881, **(K)** Unigene0042703 and **(L)** Unigene0046396.

Up-regulated differentially expressed genes, such as ABA response component binding factor, phosphatidylinositol phosphate 5-kinase, snRK2, glutamine synthetase, 90 kDa β-heat shock protein, pyrroline-5-carboxylate reductase, glutathione synthetase, serine/threonine-protein kinase, glutathione reductase, calmodulin, phosphoinositide 4-kinase A, and ETR were selected to assess the transcriptomic sequencing results with qRT-PCR. In particular, the ABA response component binding factor and snRK2 are associated with ABA metabolism. The results showed that most genes were significantly up-regulated within 8 h during drought stress. Between 8 and 24 h, 9 genes were found up-regulated and 3 down-regulated (**Figure 13**). The 12 genes were clustered into two categories, among which Unigene0036685, Unigene0033103 and Unigene0042703 belong to one category (**Figure 6**).

## Discussion

Drought stress is a major limiting factor for proper plant growth and development, especially crop production (Liu et al., 2015; Verma et al., 2021; Verma et al., 2022). It affects various physiological and biochemical activities, i.e., photosynthetic CO_2_ assimilation rate, chlorophyll synthesis, and nutritional metabolism (Li et al., 2011; Osakabe et al., 2014). Plants rapidly accumulate small molecules that help adjust osmotic response to water deficient conditions (Verma et al., 2021). Reduction in cells’ water potential enhances cells’ efficiency in absorbing and retaining water. It also maintains the swelling pressure of cells and ensures the proper functioning of plant physiological processes (Blum, 2017). Plants have a physiological adaptive strategy to respond to variations in soil moisture (%) and resistance to drought-stress conditions (Farooq et al., 2009). Osmotic adjustment is an essential physiological adaptive strategy of plants subjected to stress (Verma et al., 2022). Overproduction of ROS accumulation may cause oxidative injury at the cellular level, damage cell membranes, and lead to enzyme inactivation, degradation of protein, and cell ion disturbances (Tarchoune et al., 2010). Plants can eliminate the excess production of ROS in cells *via* enzymatic and non-enzymatic antioxidative systems (Rao and Chaitanya, 2016; Li et al., 2022).

The transcriptomic sequencing approaches are widely applied to assess plant metabolic pathways, identify new transcripts, enhance genome annotation, and screen for specific functional genes during stress conditions (Zhang et al., 2018; Felix, 2019). In the present study, there were significant changes in *S. spontaneum* transcript levels during drought stress. Pathway annotation revealed that the DEGs were mainly enriched in plant hormone signal transduction, alanine, aspartate, glutamate metabolism, protein processing in the endoplasmic reticulum, nitrogen metabolism, ABC transporters, plant-pathogen interaction, and pyrimidine metabolism. These findings verified the transcriptomic analysis results of sugarcane during osmotic stress (Santana et al., 2017). Most of the genes were significantly upregulated at 8 h after drought stress, suggesting that stress stimulated *S. spontaneum* sterols to protect cells and balance normal photosynthetic responses. Similarly, the transcriptome responses reported that the *phosphoesterase* gene was upregulated and significantly impacted sugarcane stressed plants. Plant responses begin with stress situations at the cellular level *via* activation of signal transduction pathways (Wang et al., 2020; Li et al., 2022).

The perception of water stress can trigger the activation of abscisic acid (ABA)-dependent and ABA-independent regulatory systems that govern drought-inducible gene expression (Shinozaki and Yamaguchi-Shinozaki, 2007). Genes in response to the drought-stress can be differentiated into two groups based on their functions (Shinozaki and Yamaguchi-Shinozaki, 1997; Yamaguchi-Shinozaki and Shinozaki, 2006). The first group of gene products are involved in protecting cells and regulating the signal transduction pathways of stress responses. LEA genes have been reported to protect cell structures from the effects of water loss (Bray, 1993). The LEA gene products are proposed to be located in the cytoplasm and have several features, such as hydrophilism, biased in amino acid composition, and lacking of Cys and Trp. They mainly function in the sequestration of ions, protection of other proteins or membranes, and renaturation of unfolded proteins (Bray, 1993).

Genes encoding heat shock proteins (HSPs) have also been identified to contribute drought-stress tolerance plants (Shinozaki and Yamaguchi-Shinozaki, 2007). In different plant organs of the Indian sugarcane variety (CoS 767), genes encoding HSPs are shown to be up-regulated in response to drought stress (Gupta et al., 2010). In addition, genes encoding ribosomal proteins and putative disease-resistance proteins are also identified to be up-regulated in sugarcane plant organs subjected to drought stress conditions (Gupta et al., 2010). Like ribosomal protein and disease resistance protein, it is difficult to identify the regulation of genes encoding the major water channel proteins – aquaporins – in plant leaves during stress. Aquaporins are water transporter proteins essential in adjusting or maintaining the water status during adverse environmental variables (Luu and Maurel, 2005). Other gene products associated with signal transduction, such as lipid-transfer proteins and LHCB proteins, have also been reported to be induced by drought stress in commercial sugarcane varieties (Rodrigues et al., 2009; Rodrigues et al., 2011).

Our study agreed on the expression changes of some known gene families in *S. spontaneum* in response to polyethylene glycol- 6000 stimulated drought stress. A total of 57,036,239 bases were assembled in the experiment, and 62,988 genes were obtained (**Table S3**). The unigenes have coverage distribution on reads of 1-1000 and above in *S. spontaneum*. Unigenes covering 11-100 reads were at 23216, followed by unigenes covering more than 1000 reads at 6431. Comprehensive analysis shows that the quality of the completed assembly was good. These possible novel genes were identified and required more experiments to explore. Further, their functions in water resistance in *S. spontaneum* are still unknown and need to be studied.

## Conclusion

In conclusion, we performed the combination of transcriptomic sequencing and differential expression analyses to identify pathways and genes altered under drought stress. Several physiological traits showed various trends, and transcripts in various metabolic processes reacted rapidly and strongly in response to drought stress. Genes in pathways of phenylpropanoid biosynthesis and biosynthesis of amino acids were greatly activated, and they may play important roles in stress tolerance in roots of *S. spontaneum*. Genes associated with ABA synthesis; ABA and solute transport were up-regulated during early drought stress in plant root tips. The differently expressed genes of 56237 (T_4_), 59319 (T_8_), and 58583 (T_24_), among which CK obtained the most significant number of expressed genes (35920) as compared to T_24_, with a total of 53683 trend genes. Our research is the first to explore the molecular mechanism and provide valuable information on differentially expressed metabolic pathways and genes of *S. spontaneum* in response to drought stress. This transcriptomic data will also stand as an excellent resource to explore drought stressed genes and assist in sustainable sugarcane improvement in years to come.

## Data availability statement

The original contributions presented in the study are publicly available. This data can be found at NCBI, accession number PRJNA835339.

## Author contributions

K-CW, C-MH, KV, and LX conduct the experiment. Z-ND, TP, and H-QC made statistical analyses. H-RH, H-QC, H-BL, and S-LJ participated in the analytical analysis. K-CW, C-MH, and KV wrote the first draft of the manuscript. All authors contributed to the article and approved the submitted version.

## Funding

This work was financially supported by the National Natural Science Foundation of China (32060468, 31400281) and Guangxi Natural Science Foundation (2020GXNSFBA159024; 2020GXNSFAA259061).

## Acknowledgments

We are grateful to Guangxi Academy of Agricultural Sciences, Nanning, Guangxi, China, for providing the necessary facilities and Guangzhou Genedenovo Biotechnology Co., Ltd. to assist in sequencing and bioinformatics analysis.

## Conflict of interest

The authors declare that the research was conducted in the absence of any commercial or financial relationships that could be construed as a potential conflict of interest.

## Publisher’s note

All claims expressed in this article are solely those of the authors and do not necessarily represent those of their affiliated organizations, or those of the publisher, the editors and the reviewers. Any product that may be evaluated in this article, or claim that may be made by its manufacturer, is not guaranteed or endorsed by the publisher.
