# Supplementary material for: Transcriptomic responses of Saccharum spontaneum roots in response to polyethylene glycol – 6000 stimulated drought stress
Source: Front Plant Sci. 2022 Oct 24;13:992755. doi: 10.3389/fpls.2022.992755 (PMC9638123; doi:10.3389/fpls.2022.992755)
Supplement: Supplementary file 1 [file DataSheet_1.doc]

**Supplementary materials**

**Figure S1:** Experimental flow of RNA sequencing.


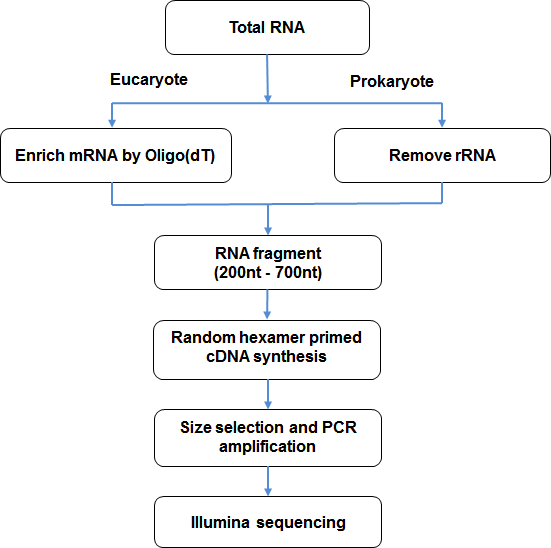


**Figure S2:** Overview of Trinity (Grabherr et al. 2011). (a) Inchworm assembles the read data set (short black lines, top) by greedily searching for paths in a k-mer graph (middle), resulting in a collection of linear contigs (color lines, bottom), with each *k*-mer present only once in the contigs, (b) Chrysalis pools contigs (colored lines) if they share at least one *k*-1-mer and if reads span the junction between contigs, and then it builds individual de Bruijn graphs from each pool, (c) Butterfly takes each de Bruijn graph from Chrysalis (top), and trims spurious edges and compacts linear paths (middle). Reconciles the graph with reads (dashed colored arrows, bottom) and pairs (not shown), and outputs one linear sequence for each splice form and/or paralogous transcript represented in the graph (bottom, colored sequences).

**
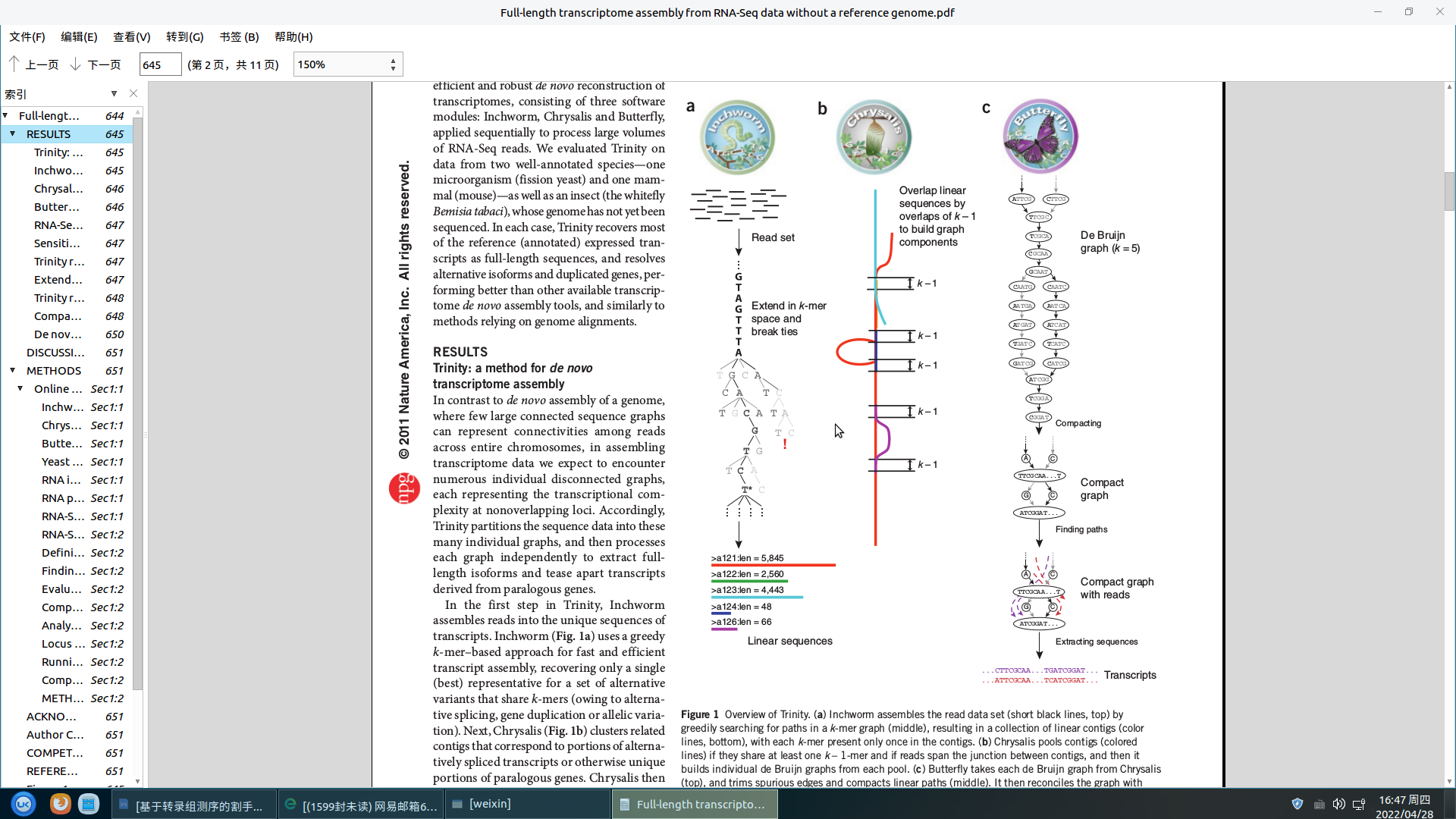
**

**Figure S3:** Steps of RNA sequencing data assembly.


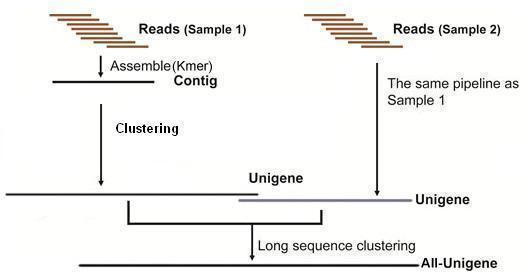


**Figure S4:** Detection map of five RNA samples.

| 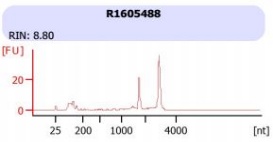 | 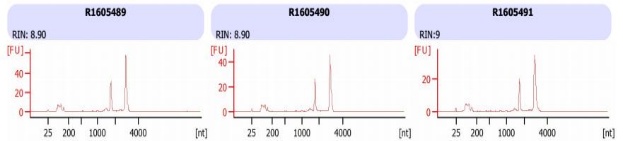 | 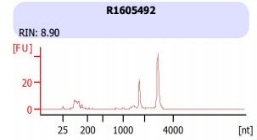 |
| --- | --- | --- |

**Figure S5:** Abundance distribution of gene expression.


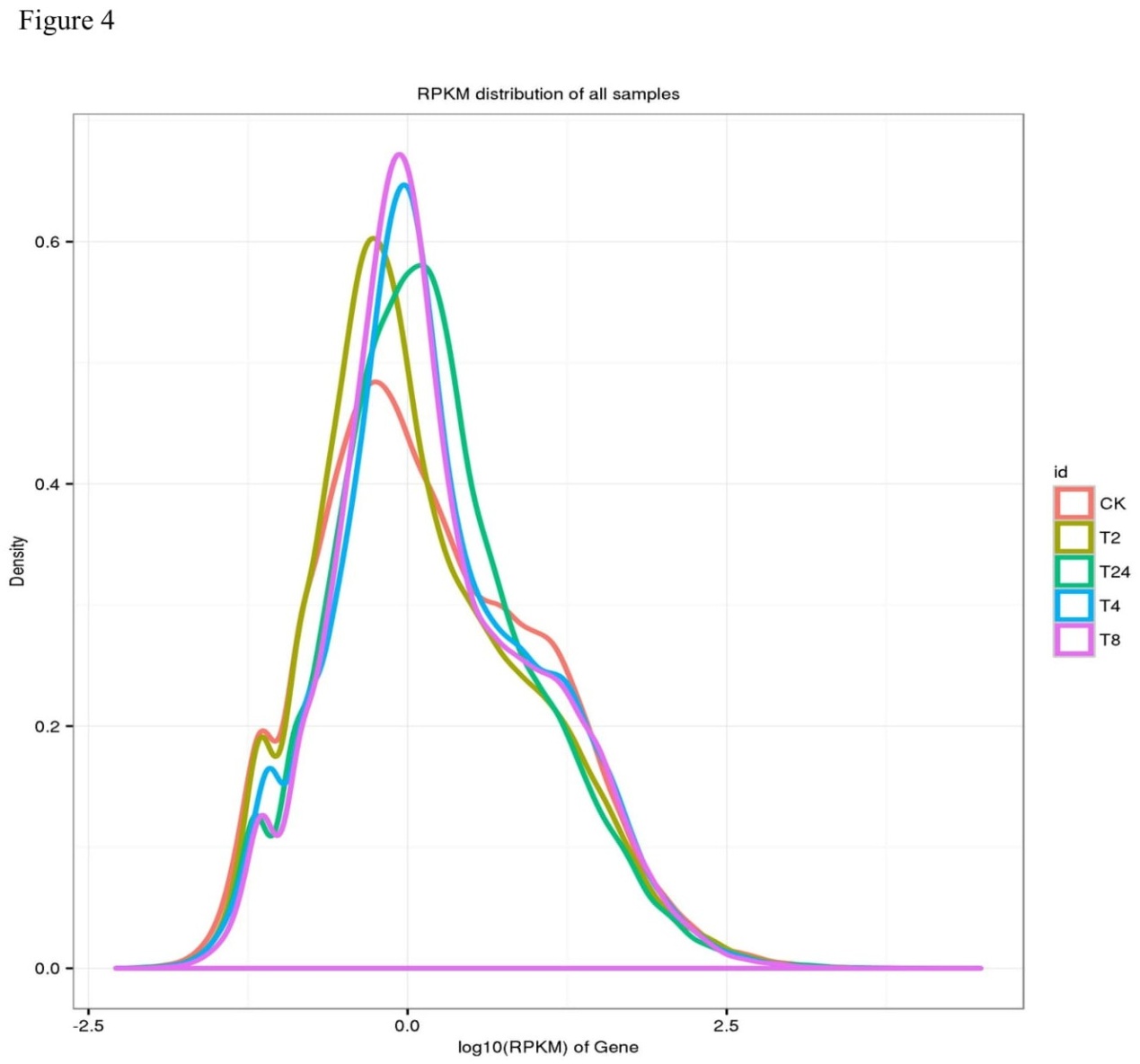


**Figure S6:** Clustering diagrams of the relationships among six samples.

**
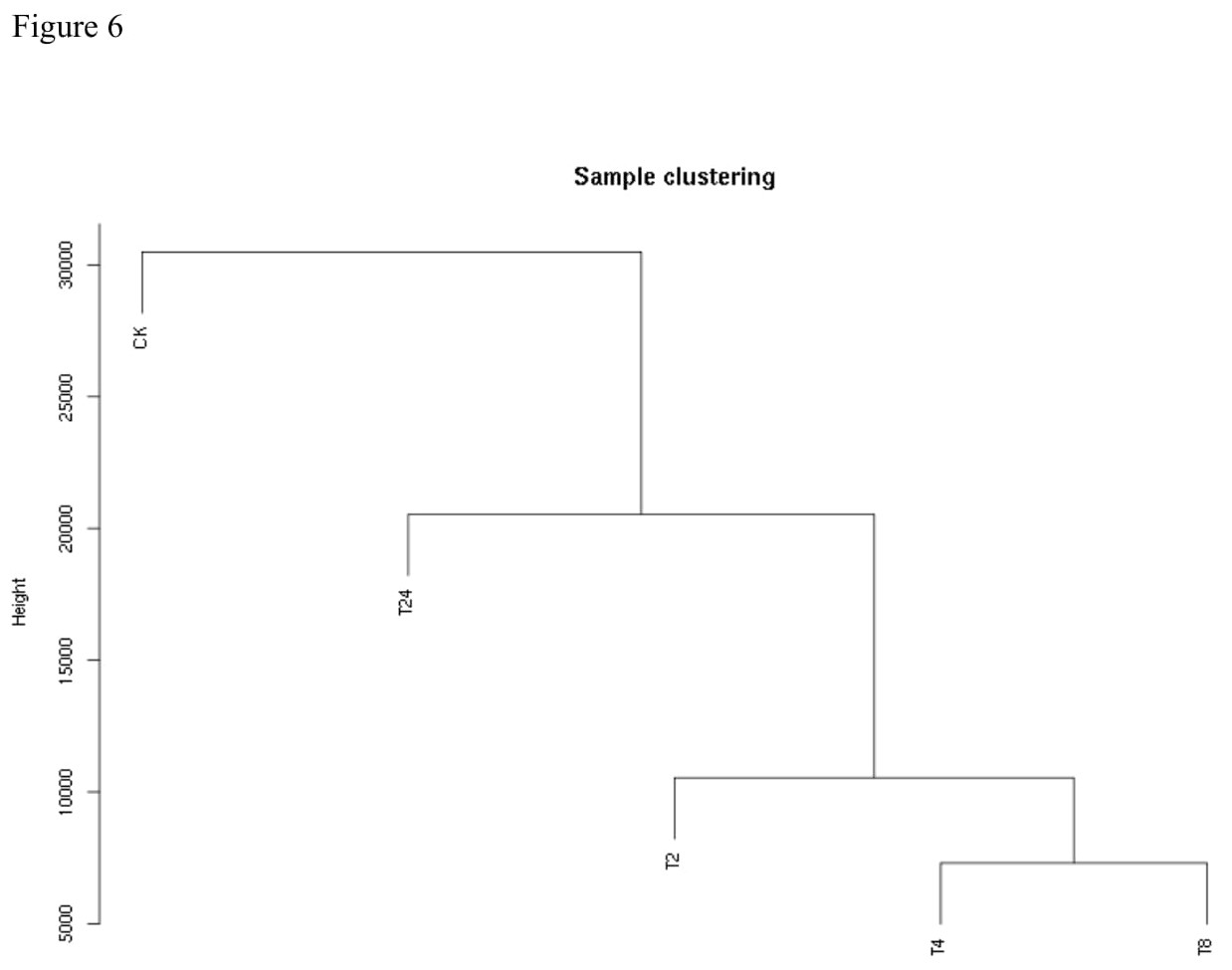
**

**Figure 7S:** Scatter plot of difference analysis between samples such as [(A) CK-vs-T_2_, (B) CK-vs-T_4_, (C) CK-vs-T_8_, (D) CK-vs-T_24_, (E) T_2_-vs-T_4_, (F) T_2_-vs-T_8_, (G) T_2_-vs-T_24_, (H) T_4_-vs-T_8_, (I) T_4_-vs-T_24_ and (J) T_8_-vsT_24_].


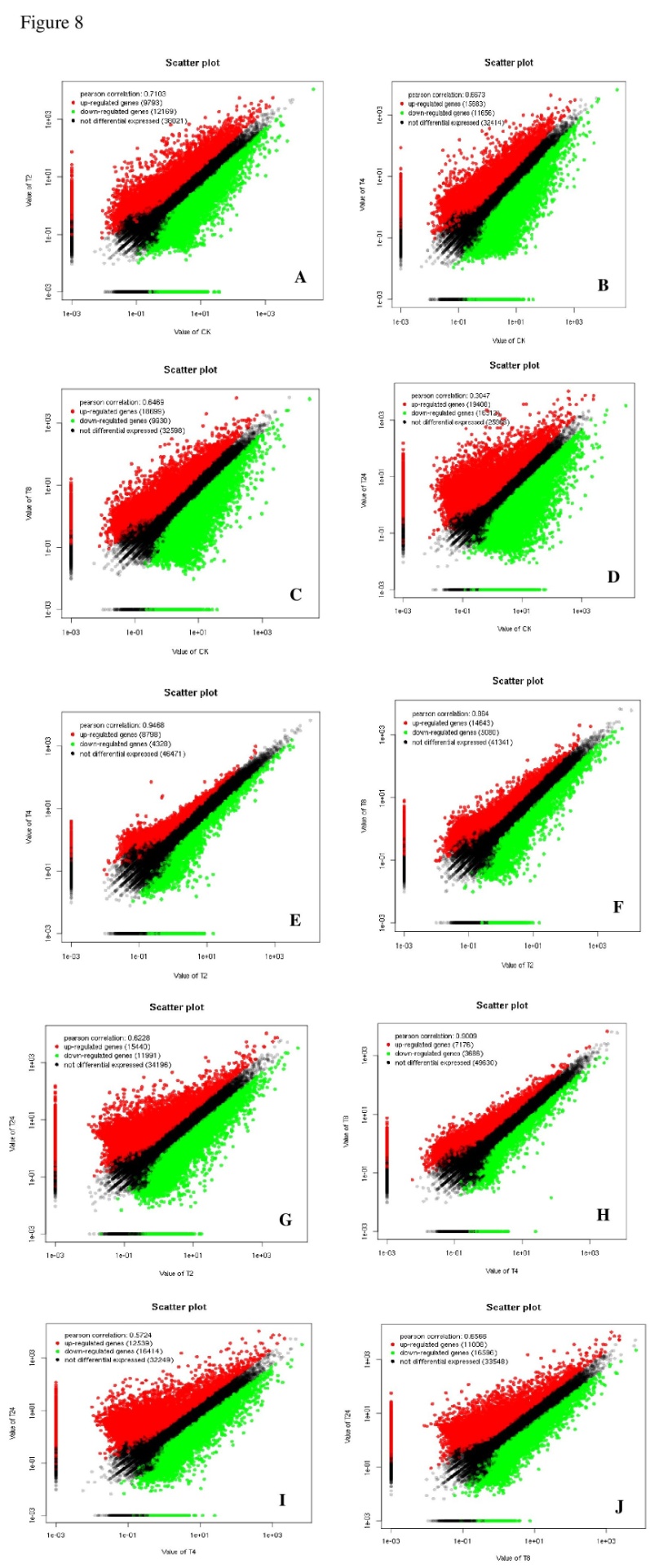


**Figure S8:** Pooled graph of gene trend lines (ID and number of genes above-trend blocks; trend blocks with color are significantly enriched trends (p-value < 0.05), trend blocks without color are non-significantly enriched trends)


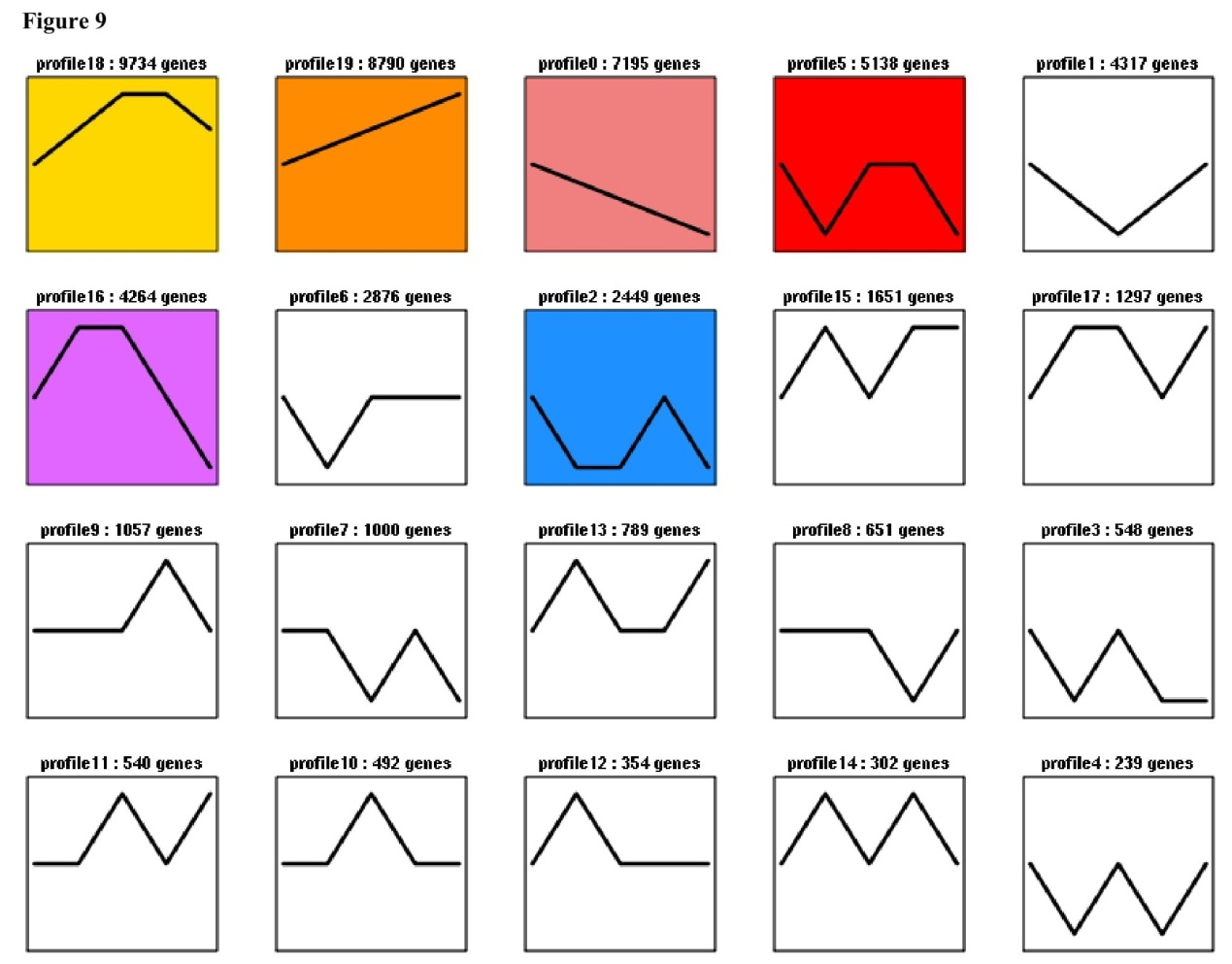


**Figure S9:** The trend gene expression profiles as (A) profile18, (B) profile19, (C) profile0, (D) profile5, (E) profile16 and (F) profile2.

**
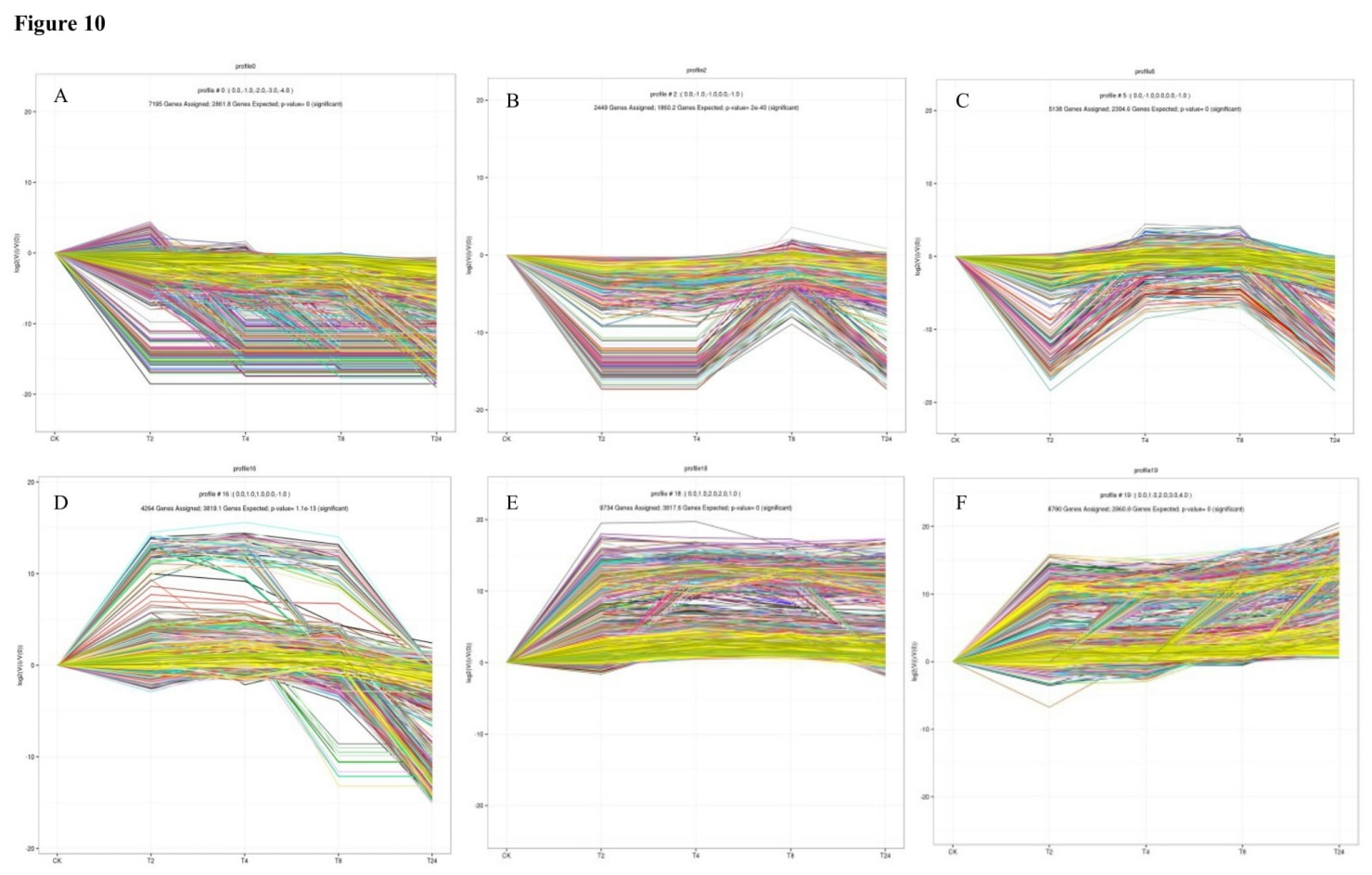
**

**Table S1:** The details of the primers used for qRT-PCR analysis

| Unigene ID | Gene function description | Primer 5'--3' |
| --- | --- | --- |
| Unigene0049197 | alpha-tubulin | F: CTCATCCCTGACTGTGTCCCTA; R: GATTTCAGCCACGGACAGC |
| Unigene0038302 | glutathione synthetase, | F: GAGGACATTGCCAAACTACGC; R: TGTTGTTCCCTCCGCCTTC |
| Unigene0011154 | glutathione reductase, | F: GGCTTTGGGTGGGTGTATG; R: GGGATGTTTGGTTTGGACG |
| Unigene0046396 | glutamine synthetase | F:AAGGATTCGGTGGATGAGTATGT; R: TGGTTTTCGGCACCCCTA |
| Unigene0038820 | pyrroline-5-carboxylate reductase | F: CCAACGGCAAAGGAAATCA; R: AGGTGGGGTCTACTCCTGTTTAT |
| Unigene0040881 | heat shock protein 90kDa beta | F: CTTTTCGCCTGATAACCCG; R: GAGGACCATTTGCCAGCG |
| Unigene0038895 | calmodulin | F: TCCGCCGCCATACTTTGA; R: GGTCATCCCTGGCTTGAATAG |
| Unigene0004788 | phosphatidylinositol 4−kinase A | F: CGAGGTGAACGGAAGGGTA; R: CCTCCAGTCACGCTTTCTAATC |
| Unigene0033103 | 1−phosphatidylinositol−4−phosphate 5−kinase | F: GGAAGGATTACTGCCCAAAGG; R: TCATCTCCACAGAGGGACAACA |
| Unigene0036685 | ABA responsive element binding factor | F: AATGGGTTGGTGTCGGGTG; R: TCTCAACCACCTTCTCCATAGC |
| Unigene0004575 | serine/threonine−protein kinase | F: TAAAGTGGCGGACTATGACCTG; R: GCTAATCTTCAGGAACCCAACA |
| Unigene0042703 | snRK2 | F: TGTCGCCAACTCGCCATT; R: GCTCCTTGGTCCGCTTGT |
| Unigene0000718 | ethylene receptor（ETR） | F: CTCATACAGTTTGGTGCGTTCA; R: CACTTTCGCCACCGTCAA |

**Table S2:** RNA samples test results

| **Test number** | **sample** | **Concentration (ng/µl)** | **Volume (µl)** | **Total (µg)** | **RIN value** |
| --- | --- | --- | --- | --- | --- |
| R1605488 | CK | 254 | 36 | 9.14 | 8.8 |
| R1605489 | T_2_ | 378 | 36 | 13.61 | 8.9 |
| R1605490 | T_4_ | 287 | 36 | 10.33 | 8.9 |
| R1605491 | T_8_ | 249 | 34 | 8.47 | 9.0 |
| R1605492 | T_24_ | 302 | 34 | 10.27 | 8.9 |

**Table S3:** Assembly quality result statistics

| Genes Num | N50（nt） | Max length（nt） | Min length（nt） | Average length（nt） | Total assembled bases |
| --- | --- | --- | --- | --- | --- |
| 62988 | 1533 | 15429 | 201 | 905 | 57036239 |
